# A Systematic Review of Head Impacts and Acceleration Associated with Soccer

**DOI:** 10.3390/ijerph19095488

**Published:** 2022-05-01

**Authors:** Ioannis Basinas, Damien M. McElvenny, Neil Pearce, Valentina Gallo, John W. Cherrie

**Affiliations:** 1 Institute of Occupational Medicine, Research Avenue North, Edinburgh EH14 4AP, UK; ioannis.basinas@manchester.ac.uk (I.B.); damien.mcelvenny@iom-world.org (D.M.M.); 2 Division of Population Health, Health Services Research & Primary Care, Centre for Occupational and Environmental Health, University of Manchester, Oxford Road, Manchester M13 9PL, UK; 3 Faculty of Epidemiology and Population Health, London School of Hygiene & Tropical Medicine, London WC1E 7HT, UK; neil.pearce@lshtm.ac.uk; 4 Campus Fryslân, University of Groningen, 8911 CE Leeuwarden, The Netherlands; v.gallo@rug.nl; 5 Institute of Biological Chemistry, Biophysics and Bioengineering, Heriot-Watt University, Edinburgh EH14 4AS, UK

**Keywords:** soccer, association football, epidemiology, peak linear acceleration (PLA), mild traumatic brain injuries (mTBIs), repetitive sub-concussive head impacts (RSHIs), sex, age, playing position, heading

## Abstract

Epidemiological studies of the neurological health of former professional soccer players are being undertaken to identify whether heading the ball is a risk factor for disease or premature death. A quantitative estimate of exposure to repeated sub-concussive head impacts would provide an opportunity to investigate possible exposure-response relationships. However, it is unclear how to formulate an appropriate exposure metric within the context of epidemiological studies. We have carried out a systematic review of the scientific literature to identify the factors that determine the magnitude of head impact acceleration during experiments and from observations during playing or training for soccer, up to the end of November 2021. Data were extracted from 33 experimental and 27 observational studies from male and female amateur players including both adults and children. There was a high correlation between peak linear and angular accelerations in the observational studies (*p* < 0.001) although the correlation was lower for the experimental data. We chose to rely on an analysis of maximum or peak linear acceleration for this review. Differences in measurement methodology were identified as important determinants of measured acceleration, and we concluded that only data from accelerometers fixed to the head provided reliable information about the magnitude of head acceleration from soccer-related impacts. Exposures differed between men and women and between children and adults, with women on average experiencing higher acceleration but less frequent impacts. Playing position appears to have some influence on the number of heading impacts but less so on the magnitude of the head acceleration. Head-to-head collisions result in high levels of exposure and thus probably risk causing a concussion. We concluded, in the absence of evidence to the contrary, that estimates of the cumulative number of heading impacts over a playing career should be used as the main exposure metric in epidemiological studies of professional players.

## 1. Introduction

During play and training, soccer (also known as association football or just football) players experience repeated head impacts from contact with the ball, other players, the ground or objects such as goal posts. These cause the brain to move rapidly within the skull, potentially creating chemical changes and sometimes stretching and damaging brain cells [1]. In some circumstances, these impacts can cause concussion, which is defined according to the Berlin Expert Panel as an “alteration in brain function, caused by an external force”. Several symptoms may be used to clinically diagnose concussive head injuries. Symptoms such as the loss of consciousness (which is not a requirement for a diagnosis of concussion) may include sequelae that result in a range of physical, cognitive, emotional and sleep-related symptoms [2], which may persist for weeks. However, in most cases the impacts experienced during soccer play result in few or no acute symptoms, and these are often referred to as sub-concussive head impacts [3] or repetitive sub-concussive head impacts (RSHIs).

Chronic traumatic encephalopathy (CTE), a relatively newly characterised pathological entity that is diagnosed at autopsy, has been linked with repeated head trauma and/or concussion experienced by boxers [4] and American footballers [5]. There have been reports of CTE in a small number of professional soccer players [6]. However, the pathological characterisation of the condition remains at a preliminary stage [7]. There is some evidence that mild traumatic brain injuries (mTBI) from playing sport can accelerate cognitive decline [8,9], and other neurodegenerative outcomes [10]. However, the long-term effects of sport-related mTBI is not well understood [11,12]. Recently, a study found mortality from neuro-degenerative diseases in former professional footballers in Scotland to be over three times that of population controls, although this contained no quantitative estimates of exposures to heading [13]; and further analysis of the data revealed the risk to be higher in footballers who played as de-fenders compared with other playing positions [14]. Currently, there is very little robust evidence from longitudinal epidemiological studies of clinical neurodegenerative disease in former professional soccer players who were exposed to RSHIs from heading balls and, occasionally, concussions. Thus, longitudinal epidemiological studies are needed to clarify these issues, ideally of a prospective design. However, since such studies on current players have only recently been established [15] it is important to also explore the presence of any effects and their association with exposure among former players. For this, retrospective study designs incorporating an objective exposure assessment are needed.

There is no clear evidence of the best exposure metric to assess the long-term risk from RSHIs and/or concussion in soccer players, although it is likely, as for many occupational exposures, that the cumulative exposure over all or part of life would be appropriate [16]. The magnitude of head impacts in soccer are generally characterised using accelerometers attached to the head that record both linear (expressed as m/s^2^ or sometimes relative to the acceleration due to gravity ‘g’ = 9.81 m/s^2^) and angular acceleration (rads/s^2^) or angular velocity (rads/s). Different methods and measurement strategies have been used to characterise acceleration, both during play and in the laboratory [17]. In addition, the number of impacts during a period are important in determining cumulative exposure [18]. However, it is uncertain whether factors such as the interval between impacts, reflecting the recovery time, or the age of the player should be incorporated into an exposure metric. It is known from neuroimaging studies that recovery time is very important for concussions [19], although its importance for RSHIs is less clear.

Quantitative retrospective estimates of cumulative exposure will require the development of a conceptual and/or empirical model that could account for the total number, frequency and types of head impacts, and secular changes over time in the professional soccer game, e.g., changes in the design of the ball, and speed of the game, as has been done for other areas of occupational epidemiology [20]. The development of such a model will require good knowledge of the factors potentially affecting exposure, i.e., the so called “exposure determinants”, but databases of exposure measurements that could support such a development are not currently available. In addition, previous review studies of the published literature in acceleration from heading have been primarily descriptive [21,22] or were performed on an ad hoc basis aiming, for example, either to evaluate the magnitude of related impacts across different sports [23] or to discuss the effects of single factors such as neck strength [24] or age [25]. 

The aim of this paper is to systematically collate, review and analyse the published scientific evidence on factors determining the magnitude of head impact acceleration, both linear and angular, during experiments and the practice and playing of the game of soccer to inform the assessment of exposure in retrospective epidemiological studies of current and former professional players.

## 2. Materials and Methods

### 2.1. Literature Searches

We identified relevant research papers reporting measured acceleration values from impacts to the head during experiments, soccer play and training. Initially our searches focused on papers published in PubMed and Web of Science indexed periodicals. However, for reasons of completeness, these searches were then expanded further to include any relevant papers published in Scopus and SPORTDiscus databases. Searches were performed according to the following search string: (“soccer” OR “football”) AND (“head impact” OR “heading” OR “header”) AND (“acceleration*” OR “instrumentation” OR “biomechanics” OR “accelerometer”). 

Restrictions on period of publication during searches were applied only according to the date the search was performed (i.e., prior to December 2021). Results were further supplemented from additional references obtained through the reference lists in the identified publications and personal knowledge of the field. To be included in the review, papers needed to:

(a) Concern the game of association football;
(b) Report original measurement results of linear and/or angular acceleration during soccer play or practice; or
(c) Be of either observational or experimental design (simulation) involving humans in realistic scenarios of play.

Papers were excluded if they were:

(a) Not written in English;
(b) Did not include original measurement data on head acceleration or did not adequately report the type of measurements they performed;
(c) Were theoretical or experimental simulations of head impacts with no human involvement;
(d) Were studies that involved humans but measured head acceleration solely on scenarios involving a pendulum; or
(e) Were conference abstracts, commentaries, or literature reviews, (although for the latter included reference lists were screened to identify further relevant studies).

### 2.2. Review Process and Data Extraction

Following retrieval of the search results, the identified papers were screened by title and abstracts against the above criteria. Papers that could not be clearly excluded by the above criteria were retained for full text evaluation. For papers not excluded during this process, the full text was retrieved and evaluated. Identified studies that fulfilled the above criteria were reviewed in full and had their data extracted.

Data extraction was carried out using a dedicated MS Excel template with separate spreadsheets for storing data related to the measurements of acceleration, the frequency of head impacts and the reported results by the identified studies on the effects of potential determinants, i.e., factors affecting the intensity and frequency of head impacts. Extracted parameters included: the manuscript reference (authors, title, publication year); the applied design (observational or experimental); the population characteristics (e.g., gender, and age); a description of applied measurement methods including of any applied threshold; a description of the scenario and activity considered; the number of measurements performed and head impacts experienced; and the results of the measurements of head linear and angular acceleration. The spreadsheet containing the reported information on the frequency of the head impacts observed during play covered only observational studies and included data on previously mentioned study design, population characteristics and measurement data stratified by playing position. For exposure determinants, registered parameters included general information on the study and its design (i.e., observational or experimental), the name of the examined determinant/s, the outcome investigated (i.e., peak linear and/or angular acceleration, head impact frequency), the methods used to analyse the collected data and the result of the analyses undertaken.

Paper reviewing and data extraction was performed by one experienced human exposure scientist (IB). Quality control was provided by a second human exposure scientist (JWC) who independently reviewed and extracted the data for 10% of the selected papers (including papers regarded as more complex). The extracted data from both reviewers were compared, and disagreements discussed and resolved.

Existing criteria for the evaluation of observational epidemiological studies or studies in sports are not relevant for the evaluation of occupational exposure studies whereas dedicated formal criteria for evaluating such studies are at present also not available. To evaluate the quality of the individual studies included and the risk of bias we therefore created and implemented an ad hoc set of quality criteria that considered attributes of the monitoring study design (adequate description and potential bias), population representativeness, and the reporting of the characteristics of the population (e.g., age, sex, experience), measurement methods, sample size, the distribution of the acceleration measurements and whether or not impacts have been confirmed. Each of these attributes was scored depending on whether the attribute was present or absent on a scale between 1 and −1, respectively. Only design parameters relevant to the acceleration measurements were evaluated in this process. The complete matrix of the attributes, their characteristics and the scores assigned are summarised in the online supplement (Supplementary Table S1). The assigned score results were accumulated and their total used to assess the overall quality of the included studies. Studies were considered as of high quality if they had achieved a total score of ≥6, of medium quality if they their score was between 4 and 5 inclusive and of low quality if their total score was ≤3.

### 2.3. Data Rectification

Whenever required the provided linear and angular acceleration results were standardised to the relevant SI units (i.e., m/s^2^ and rad/s^2^). Summarised statistics for these metrics extracted from the papers included the reported number of measurements in the series, the arithmetic (AM) and geometric mean (GM), the range or selected percentiles of the distribution of measurements and the standard (SD) or geometric standard deviations (GSD). Whenever the AM, GM and GSD values were not available, where possible, these were calculated from the information available using the equations previously provided by Lavoue et al. [26]. For frequency of head impacts three different statistics were derived and/or, whenever possible, calculated by the information available: (a) number of head impacts per activity, (b) average number of head impacts per player per activity, and (c) average number of head impacts per hour of play per player. Age of player was defined as being of early youth (<14 yrs old), youth (15–21 yrs) and adult (>21 yrs), or mixtures of these categories. Assignment of records to these categories was based on the reported mean age of the players participating in the corresponding measurement series. 

### 2.4. Data Analysis

Statistical analyses were carried out using Stata 17 (Statacorp, 2021. Stata Statistical Software: Release 17. College Station, TX, USA: Statacorp LLC.). All analyses were based on GM values. Comparisons between factors potentially influencing the magnitude of linear and angular acceleration during head impacts were performed using standard statistical approaches. These included graphical representation and statistical tests were carried out based on *t*-tests based on simple linear regression with no constant. The relationship between linear and angular acceleration was explored using weighted pairwise Pearson correlation coefficients.

## 3. Results

**Figure 1:**
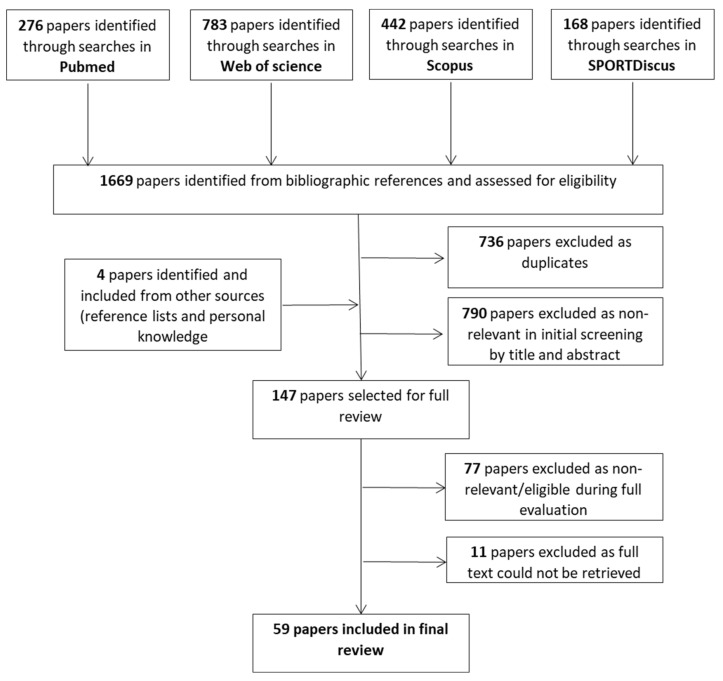
The literature search and review process.

A schematic representation of the search results and review process is shown in Figure 1. Overall searches resulted in 1059 candidate papers identified. Following removal of duplicates and screening by title and abstract 119 papers were retrieved and read in full resulting in 59 papers that fulfilled the criteria and were included in the final review. From those studies, 26 followed an observational design, 32 were experimental studies and one had both an experimental and observational component.

Table 1 contains the characteristics of the 27 observational studies included in the review, the earliest of which was published in 2012. Eight studies investigated early youth players (i.e., ≤14 yrs old), 18 studied youth players (i.e., aged between 15–21 yrs old), and one included a mixture of youth and adult players. Twenty-one studies included measurements on females, twelve on males and one study reported its results without stratification by sex. Most of the studies (*n* = 13) used accelerometers mounted on the side of the head somewhere behind the ear, four used customised headbands, six used a customised mouth guard and one each used accelerometers mounted on the back of the head or in the ear. Some studies (*n* = 17) used observation of play (either through video recordings or by observing the actual game) to confirm whether the accelerometer data were linked to heading or another external impact event.

Table 2 contains the characteristics of the 33 experimental studies. Examined scenarios varied considerably between these studies. Seventeen of the studies measured acceleration in youth players, nine in adults and another seven combined players of different ages. Seventeen studies measured acceleration in males, two in females, thirteen in both males and females, reporting the results either individually for the two sexes (*n* = 4) or combined (*n* = 9), and one did not specify the sex of the participants. The locations of the accelerometers for the experimental studies were much more varied than for the observational studies. A customised mouth guard was used in eight studies. Two used an accelerometer held in place with an elastic skull cap, seven had the sensor mounted on a headband, three behind the ear, another on a helmet and another on an unspecified location on the outer head. One study used multiple approaches including customised mouth guards and sensors mounted behind the ear and in an elastic skull cap. Two studies used a bite plate, one had sensors mounted in various places on manikins, two studies located the sensor in the ear, three on the base of the skull and two studies used motion capture with video camera data.

**Figure 2:**
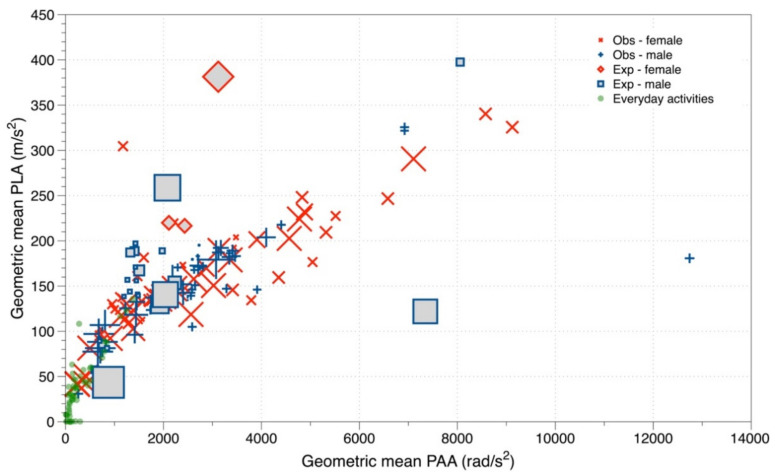
Scatter plot of mean peak linear and angular acceleration, by experimental (squares and diamonds) and observational (cross and plus) studies. Symbol area reflects the number of measurements associated with the mean.

Mean peak linear and angular acceleration (PLA, PAA) varied widely between studies. The geometric means for impacts during observational studies of play or training ranged from around 30 m/s^2^ and 240 rad/s^2^ to around 450 m/s^2^ and nearly 7000 rad/s^2^. Figure 2 shows a scatter plot of the mean PLA against the corresponding PAA values for the studies that measured both (red for female and blue male; open symbols for observational studies and boxes or diamonds for experimental studies). The data are subdivided into those from experimental studies with humans and/or manikins and observational studies involving free play or training, with the area of each symbol proportional to the number of measurements in the dataset. For comparison we have also added PLA data from everyday non-sport activities as green circles [59]. The pattern of results differs depending on the study design, although in each case there is an apparent linear relationship between both measures of acceleration (for the observational studies the pairwise correlation coefficient, weighted by the number of measurements, was 0.90, *p* < 0.001). Overall, mean PLA were on average higher for males compared to females in observational studies (172 vs. 159 m/s^2^, *p* < 0.001). Excluding the studies by Patton et al. [20,25], which include unusually high PLA data from non-header head impacts, results in the means for females being higher than those for males with PLA of 156 and 136 m/s^2^, respectively (*p* < 0.001). Because of the strong correlation between linear and angular acceleration we have restricted the remainder of the paper to report linear acceleration, although the complete data are available in the online Supplementary Tables S2 and S3. Also, because the experimental studies have a different relationship between linear and angular acceleration compared with observational studies, probably because of constraints on the way the ball was headed in the former studies, and because they should better represent the forces experienced during actual play we have restricted the summarisation of the data to observational studies.

**Figure 3:**
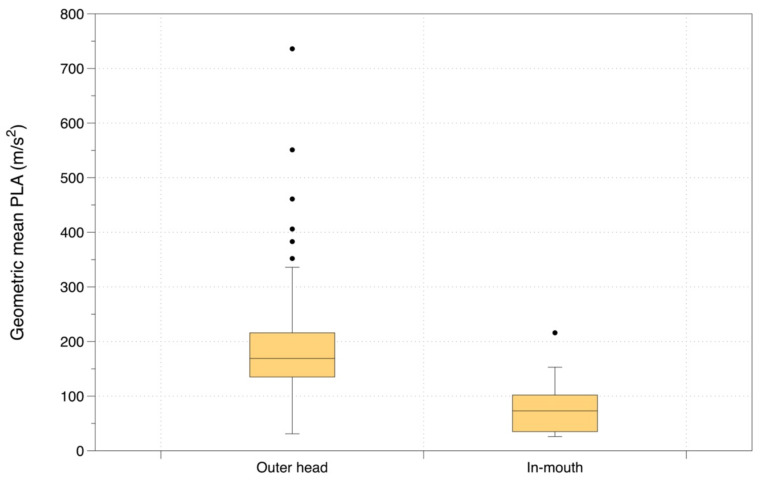
Geometric mean PLA by method of data collection for observational studies.

Figure 3 summarises the observational data by the strategy used by the researchers to locate the accelerometers on the players, grouped as mounted on the exterior head and using in-mouth sensors. Mean acceleration for the in-mouth location was 86 m/s^2^ compared to 196 m/s^2^ when the sensor was attached to the outer head; these data were significantly different (*p* < 0.001). Most of the studies used a threshold below which data were discounted, typically 10 g (98 m/s^2^), although some studies used a lower threshold (3 or 7 g) and some a higher threshold (20 g) (data not shown). The limited data available suggest that the measured PLA is strongly dependent on the choice of threshold with the median acceleration for the higher threshold being 1.8 times the median for the more typical threshold. Most studies used visual confirmation of an impact for it to be accepted as a genuine head impact rather than a spurious event recorded by the accelerometer. The PLA measured with observational confirmation were on average slightly lower than the data where impacts were unconfirmed (data not shown).

**Figure 4:**
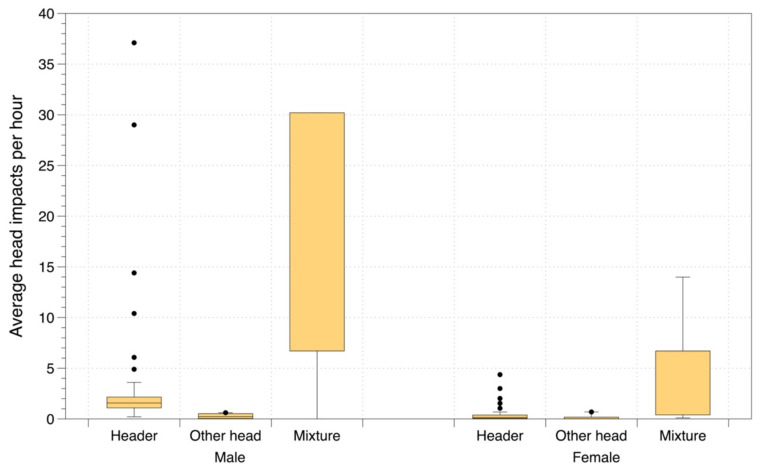
Number of head impacts per hour for male and female players, categorised by heading and mixed head impacts.

Not all the identified studies recorded the number of head impacts during a match or period of play, but for the 25 observational studies that did, we have summarised the data in Figure 4. Note there are also several studies that recorded the number of head impacts in soccer but did not measure acceleration of the head and therefore these studies were also not included in our review. The mean number of head impacts per hour from heading was statistically significantly higher in males than females, (2.4 vs. 0.5 per hour, *p* = 0.003) and for any other type of head impacts (6.1 vs. 1.4 per hour, *p* = 0.014). The number of impacts experienced was somewhat higher in defenders compared to the other positions, although when broken down by sex there were insufficient numbers to support any meaningful analyses (data not shown). 

**Figure 5:**
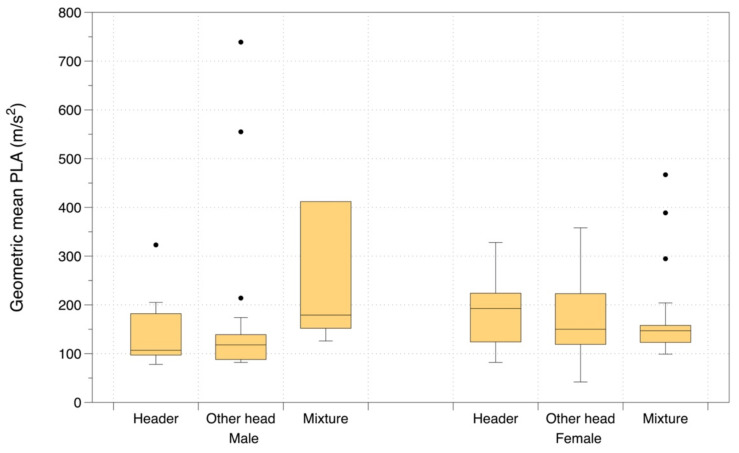
Geometric mean PLA for various types of head impact during play, for male and female players.

Figure 5 shows the data on geometric mean PLA for the type of head impact, grouped by ‘heading’, ‘any type of head impact (i.e., mixture)’ and ‘other than heading head impact’. The geometric mean linear acceleration associated was 176 m/s^2^ for headers and 169 m/s^2^ for other head impacts. The differences between the sexes for both types of head impact were statistically significant with females experiencing higher intensity impacts (*p* < 0.001).

**Figure 6:**
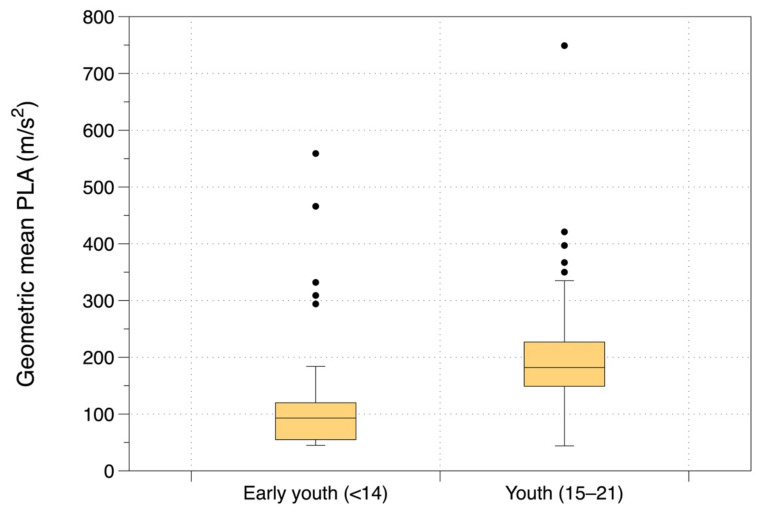
Geometric mean PLA from observational studies subdivided by age.

The observational studies covered early youth (age under 14 years) and youth players (age 15 to 21) (Figure 6). The geometric mean PLA was lower for the youngest players (early youth), 99 m/s^2^) and higher for older players (192 m/s^2^ for youth). The differences between these age groups were statistically significant (*p* < 0.001).

Table 3 summarises data from the individual studies reporting potential determinants of head acceleration and frequency associated with heading. Gender was subject of investigation in 13 studies, age in seven studies, size of head and/or neck in six, type of event in 15, playing position in six, and the type of head impact event in 20. Other, less studied, exposure determinants examined included ball characteristics and speed, heading technique, and game half.

There were suggestions that midfield players and defenders were likely to experience more head impacts than forwards and goalkeepers [6,7,10]. Similarly, one study reported somewhat lower acceleration from impacts for goal keepers compared with players in other positions [7] whereas another suggested defenders to experience significantly higher acceleration than players of other positions. Differences in PLA or PRA associated with different types of headers were also reported. While in some cases these were statistically significant these differences were rather small in absolute values. For example, Sandmo and colleagues measured the acceleration for six male youth players who completed five different heading drill exercises [18]. The highest median PLA was for finishing headers (around 320 m/s^2^) and the lowest for direct short headers (around 90 m/s^2^). The data from the included observational studies were broadly consistent with these findings. For example Lamond et al. [10] reported PLA that ranged from a median of 200 m/s^2^ for headers from passes to 290 m/s^2^ for headers regarded as shots at goal.

**Figure 7:**
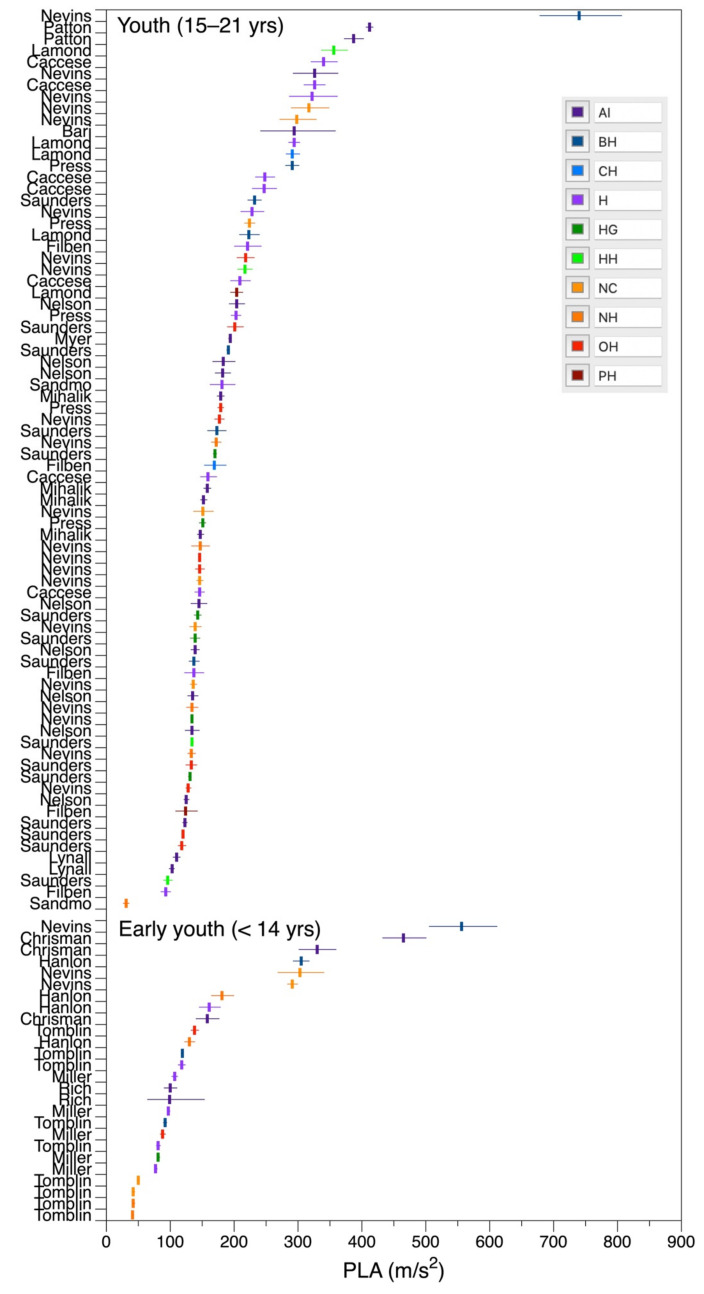
Forest plot of PLA for each observational study by activity or playing position, subdivided by early youth and youth.

Figure 7 shows a forest plot of the data from the individual observational studies subdivided by age. Each data item reflects the activities described by the authors of the studies, for example ‘HG = header to ground contact’, ‘HH = head-to-head collision’ as described in the figure caption. The figure illustrates the heterogeneity between the studies and activities, and the contrast between the ages. 

Activity: H = Header; NH = Any non-head impact; BH = Ball to head; AI = Any head impact; NC = Type of head contact not clear; HG = Head hit ground; PH = Passing header; CH = Clearing header; HH = Head to head collision; OH = Other player collided with head.

The complete results of the quality assessment of the included studies is provided in the online supplement (Supplementary Table S4). Overall, 34 studies have achieved a score ≥6, 18 a score between 4 and 5 and the remaining seven had a score ≤3. Of the 34 studies with a quality score ≥6, half were of observational design and the other half of experimental design. Note that this assessment does not account for the relative merits of the measurement methods applied (e.g., measuring with a mouth guard vs. with sensor attached over the mastoid process) which in general can also impact on the reliability of the acquired estimates to a degree such that it cannot directly reflect the exposure of professional adult players. Overall, there is little evidence for large differences in PLA by playing position, although the difference between males and females is clear. The evidence suggests children have a low frequency of heading and experience lower PLA than adults from each header.

## 4. Discussion

The accelerometers used to evaluate head impacts amongst soccer players measure both angular and linear acceleration. For the observational studies, there was a strong correlation between these two measures and therefore for the purposes of characterising exposure for retrospective epidemiological studies it is sufficient to consider just one; we have selected PLA for this purpose. Angular acceleration measurements should still form part of any exposure characterisation studies and the best exposure metric for prospective epidemiological studies may be a more complex combination of these kinematic measures [62,63]. In contrast to the observational studies, the data from experimental studies showed a different and weak correlation between linear and angular acceleration (r = 0.19, *p* < 0.001), and in general for a given angular acceleration the PLA appears to be higher in experimental than observational studies. It is not clear why there is this difference, or whether heading a ball gives rise to similar levels of linear acceleration in the necessarily constrained circumstances of experimental situations compared to free play. Clearly experimental studies are a poor proxy for normal play and thereby have limited relevance in assessing cumulative exposure over a playing career. However, data from experimental studies could provide useful data on specific aspects of heading to assess relative effects, e.g., studies of the difference in ball design or weight [33], the type of heading [18], or deliberate impacts to the head such as from shoulder-to-head or head-to-head impacts [35].

The location and fixing of the acceleration sensor to the head is an important factor in the measurement. Sensors located in a mouth guard seem to provide much lower measures of acceleration than sensors more loosely mounted on the side of the head. Sensors attached on the outer head have previously been reported to over predict exposure in terms of acceleration due to motion between the sensor and the skull during use [6,45]. The threshold for the minimum acceleration recorded also affects the data obtained, although additional visual confirmation of head impacts to exclude spurious sensor data appears to make less difference in the measurement of linear acceleration. It has been suggested that data analysis using algorithms, a common approach to remove spurious impacts from a measurement series, are currently inadequate to identify genuine head impacts during play [20]. It is important to standardise measurement methodology to obtain comparable data between studies, and we recommend that researchers use a mouth guard-mounted accelerometer or similar rigid fixing, with a threshold of 10 g for linear acceleration from individual impacts (around the maximum encountered in everyday non-sport activities). Visual confirmation will further improve the reliability of measurement data and could provide additional context for the impact that could aid data analysis.

Non-ball events, such as head-to-head contacts, can produce linear acceleration two to five times that of ball contacts and may also cause concussion. Particular emphasis should be placed on identifying and quantifying these events in measurement studies. For example, Lamond et al. [10] found that median PLA from head-to-head contacts amongst collegiate female players was 350 m/s^2^ compared to around 200 m/s^2^ during headers from passes. However, Nevins et al. [11] carried out an observational study of eight male high school soccer players over a playing season, and identified that 18% of the impacts were due to player-to-player contacts, but for these the median PLA was around half of that experienced during heading events. Others similarly found lower PLA associated with non-heading compared with heading events [18,21]. In contrast, the experimental studies involving dummy heads consistently showed higher acceleration. Hanlon et al. [64] found increasing PLA as impact speed increased in simulated head-to-head collisions, around 300 m/s^2^ at 2.5 m/s and around 700 m/s^2^ at 3.5 m/s with corresponding ball-to-head values around 150 m/s^2^ at 8 m/s impact speed. In similar experiments, Withnall et al. [35] found elbow-to-head impacts produced mean PLA of 210 m/s^2^ and hand/wrist/forearm-to-head 200 m/s^2^, and head-to-head contacts produced mean PLA of around 300 m/s^2^ at 1.5 m/s impact speed and around 800 m/s^2^ at 3 m/s.

Reported geometric mean PLA varied from around 30 to over 400 m/s^2^, although most of the measurements ranged between about 40 and 350 m/s^2^. This is a very narrow range of data when compared to other occupational exposure situations, e.g., chemical exposures [65], which probably reflects the similarity of exposure from heading and other head impacts while playing soccer. Most observational studies set a minimum threshold, typically between around 50 and 200 m/s^2^ below which data are discounted, although most commonly 98 m/s^2^ (10 g). This value is consistent with excluding the accelerations typically experienced in everyday life which are shown in Figure 2; where acceleration from individual events seldom exceeds 100 m/s^2^ or 1000 rad/s^2^ [59]. Also, there is an upper limit to the acceptable acceleration in normal play because above around 1000 m/s^2^ there is a clear risk that the player will suffer a concussion [12,66]. It is though important to note that risks at lower levels than those cannot be excluded, whereas interpretations need be cautious given the demonstrated differences in measured acceleration levels between in-mouth and outer head methodologies. Nevertheless, this relatively narrow range of exposure from individual heading events suggests that in a retrospective epidemiological study it may be sufficient to assume that on average all headers contribute equally to exposure.

It is notable that most of the studies contributing to this review were from USA and from younger non-professional players. Efforts should be made to collect acceleration measurements from professional players today and to compare these data with corresponding data from non-professionals. There are no data from play prior to around 2000, and it is possible that differences in play from these earlier times, for example the ball may have been in the air more because of the generally poorer state of playing pitches, may have affected exposures of players in the 1960s and 70s who may be those at risk of developing chronic neurological disease now. It is often anecdotally reported that the older style leather balls were more uncomfortable to head. However, it is interesting that the specification for ball size, weight and inflation pressure have remained more or less unchanged since the 19th Century, although the older leather balls were reportedly more prone to absorb water and consequently could become heavier through use. Shewchenko and colleagues [33] showed that older wet balls could increase in mass by between 3 and 47%, although the relative change in head linear acceleration in their tests was less than 20%. Given this relatively small effect we do not considerate it appropriate to attempt to adjust the head impact exposure assessment for differences in ball weight in retrospective epidemiological studies.

Women appear, on average, to have higher PLA from each head impact during play but they experience fewer impacts per hour of play than men, and so their cumulative exposures during a playing career are much lower. This has been highlighted in a number of publications from experimental investigations [37,46] and in previous reviews [67], but not in all such studies [40]. Experimental studies have demonstrated female soccer players have significantly poorer neck strength compared to male players during heading [37,55]. Other studies have confirmed that female neck muscle strength is substantially weaker than corresponding males [68]. Additionally, on average women have lower head mass [69,70], which would result in proportionately higher acceleration from the same impact force. Caccese et al. [47] demonstrated in a controlled experimental study that both estimated head mass and neck strength were significantly associated with PLA in heading; on average, PLA increased by about 50% from around 3 kg to 7 kg head mass. It seems likely that the difference in head acceleration experienced by females and males results from sex-specific differences in neck muscle strength and to a lesser extent differences in head mass.

Impact to the head may result in a range of traumatic physical and biochemical changes, including damage to the blood-brain barrier, abnormal neurometabolism, neuroinflammation along with aggregation and deposition of tau protein in the brain [71]. Some researchers have suggested that because it takes a finite amount of time for the brain to recover from a mTBI, repeated head impacts within the recovery time may have a disproportionate effect on cumulative injury. For example, in an experimental study in adult male rats given two traumatic head impacts, separated by either 24 h or 3 days [72], when the second mTBI was given during the first injury cerebral glucose metabolism recovery (CMRg) period (24 h) it prolonged the CMRg dysfunction and animal behavioural impairments compared to the longer time interval. It may take up to a week or more for symptoms to resolve following a concussion [73]. Merchant-Borna et al. [74] developed a series of exposure metrics for an epidemiological study of American Football players based on a weighting of PLA and other measures of head impact by the inverse of the time between hits and/or the time interval from hit to post-season health assessment. However, these assessments required detailed measurement of the frequency and intensity of impacts that were collected prospectively using helmet-mounted accelerometers. For the purposes of retrospective exposure assessment for studies of professional soccer players, which will inevitably rely on self-reported information on playing, there will likely be insufficient data on head impact frequency to develop this type of exposure metric.

It is also not clear how RSHIs could be linked to the biological changes in the brain following impact or long-term risk of disease, and so it is premature to try to develop an exposure metric that reflects the biological harm. However, given the nature of the potential injury, some form of lifetime cumulative exposure metric, as has been used in other sports involving repetitive head impacts [75], seems appropriate. Given the close relationship between linear and angular acceleration in soccer play and the relatively narrow range of accelerations experienced during play and training, we propose that the lifetime number of impacts would be appropriate. Data on the number of head-to-head collisions and the number of other blows to the head other than head-to-head collisions (i.e., elbow, kick and ball strikes or collision with goalpost and/or the ground) should also be collected and ideally these should be combined with the number of headers, weighted for the relative difference in PLA for these events.

There is evidence suggesting that playing position may influence the number of times an individual heads the ball, with perhaps midfield players and defenders being more likely to head the ball and goal keepers least likely to head the ball; although patterns may differ dependent on the league involved. Recent data from the English Football Association confirmed that defenders headed the ball on average 7.5 times per 90 min which was almost twice the rate of other players (average 3.6 to 4.5 per game) [76]. The rate was highest for the English Football League (League 1, 2 and the Championship) at 9 to 10 per 90 min for Defenders and lowest for younger defenders (Premier League under 23 and under 18 s) at 4 to 6 per game. Results from studies from other European leagues further support the importance of playing position as a determinant of the frequency of heading during professional or semi-professional level of play [77,78]. However, this has also been suggested to depend on age and/or level of play [79]. Despite the above, from our review the average acceleration does not appear to vary greatly by type of header and less so by position of play. While there is data in the literature that could be used to estimate the number of head impacts by playing position or era of play, we consider the best approach would be to collect such data for different periods of play and training by interview with individuals enrolled in an epidemiological study. This is important to account for variations sourcing from differences in team systems and personal style of play. Since self-reported data can be subject to potential recall bias [80], studies should include some form of validation of the former player’s recall of their heading during play would also be needed. This could likely be achieved by comparisons with historical video footage and archives of whole match sections, rather the video highlights.

There has been much discussion about the potential risks of young people heading soccer balls, and young people’s developing brains may be particularly vulnerable to repeated sub-concussive impacts [81]. Although the scientific evidence is still unclear about potential risks, some soccer authorities have decided to introduce restrictions on purposeful heading for younger players, and for example in 2020 the English Football Association required that children under 11 years should not have training in heading and heading drills should be reduced as far as possible for all players under the age of 18 years [82]. Guidance for training in heading among adult professional and amateur players have also been produced [76,82]. On average, younger children appear to have lower PLA than older youths or adults, and they generally head the ball less frequently than older players [67]. However, it is unclear what contribution heading during youth may have on the developing brain and we recommend that in epidemiological studies the contribution of repeated head impacts be examined separately for childhood and adulthood to investigate whether there is differential susceptibility to trauma.

Retrospective exposure assessment for epidemiological studies is problematic in most situations because of lack of historic data. In studying chronic neurological disease in soccer players, it is made particularly difficult because the biological mechanisms underpinning an association between mild traumatic head impacts from heading soccer balls are unclear and so the most appropriate exposure metric is uncertain. Additionally, there are no data in relation to acceleration for professional players. However, the evidence suggests that the range of head acceleration during playing soccer is generally quite small and does not vary much between playing positions. We have concluded, because of a lack of evidence to the contrary, that the best approach is to rely on estimates of the cumulative number of heading impacts over a playing career as the main exposure metric in epidemiological studies of professional players. 

## 5. Conclusions

Information about head acceleration experienced by soccer players is available from experimental and observational studies, but the latter are more informative of acceleration during actual play. There is a close association between linear and angular acceleration from the observational studies and for the purposes of informing exposure assessment in epidemiological studies it is sufficient to consider just one of the two measures. For the purpose of our review we selected peak linear acceleration (PLA) but we need to acknowledge that the relationship between the two measures may be much more complicated that a simple linear relationship. Most of the available data are from the USA and from younger non-professional players. There are substantial differences in the data depending on measurement techniques and for this reason the available data are not particularly informative; standardisation of methodology is important for future studies. However, PLA experienced by female players is on average higher than for male players and young people generally experience lower PLA than adults. The range of PLA measured in soccer play and training is relatively small compared to the differences in other types of occupational exposures, for example chemicals of dusts. Clearly, it would be helpful if more informative studies were carried out in order to allow the relevant exposure metric to be determined. These include mechanistic studies and studies of adult professional association football players. However, to estimate the head impact exposure of professional soccer players in epidemiological studies, based largely on a lack of evidence to the contrary, it is recommended to use the cumulative number of heading impacts over a playing career.

## Figures and Tables

**Table 1:** Observational studies reporting peak linear and/or rotational accelerations due to heading and other head impacts.

Reference, Country	Population	Sensor Location	Scenario	Activity/ Position	Number of Measurements/ Sport Events *
Hanlon et al., 2012 [1], USA	24 FY	OH, back of head	Training	H	>24 measurements
				NH	
				F	
				BH	
				GP	
McCuen et al., 2015 [2], USA	35 FY	OH, behind ear	Games and Training, (over season)	AI	Not specified
Caccese et al., 2016 [3], USA	16 FY	OH, custom headband	Games (over season)	VH (*n* = 6)	≤224 measurements
				UH	
Chrisman [4] et al., 2016, USA	7 FEY, 10 MEY	OH, behind ear	Games over a weekend tournament (3–6 games)	AI	72 measurements
Lynall et al., 2016 [5], USA	22FY	OH, behind ear	Games (over season)	AI	≤252 measurements
			Training (over season)		≤858 measurements
Press et al., 2017 [6], USA	26 FY	OH, behind ear	Games and Training (over season)	H	≤916 measurements
			Games and Training (over season)	NC	
			Games and Training (over season)	HG	
			Games and Training (over season)	BH	
			Games and Training (over season)	UH	
Reynolds et al., 2017 [7], USA	1 MY	OH, behind ear	Games (over season)	AI, Goalkeeper	7 measurements
	2 MY		Games (over season)	AI, Defence	15 measurements
	1 MY		Games (over season)	AI, Midfield	2 measurements
	4 MY		Training (over season)	AI, Goalkeeper	50 measurements
	2 MY		Training (over season)	AI, Defence	93 measurements
	4 MY		Training (over season)	AI, Midfield	32 measurements
	2 FY		Training (over season)	AI, Goalkeeper	42 measurements
	2 FY		Training (over season)	AI, Midfield	36 measurements
	3 FY		Training (over season)	AI, Forward	59 measurements
Reynolds et al., 2017 [8], USA	4 MY		Training (over season)	AI, Goalkeeper	115 practices
	6 MY		Training (over season)	AI, Defence	197 practices
	4 MY		Training (over season)	AI, Midfield	125 practices
	1 MY		Training (over season)	AI, Forward	43 practices
	1 MY		Games (over season)	AI, Goalkeeper	2 games
	1 MY		Games (over season)	AI, Forward	7 games
	3 MY		Games (over season)	AI, Defence	19 games
Bari et al., 2018 [9], USA	23 FY	OH, behind ear	Games and training (over 1–2 seasons)	AI	29
Lamond et al., 2018 [10], USA	23 FY	OH, custom headband	Games and training (over season)	PH	≤961 measurements
			Games and training (over season)	CH	
			Games and training (over season)	AH	
			Games and training (over season)	HH	
			Games and training (over season)	UH	
Nevins et al., 2018 [11], USA	8 MY	OH, behind ear	Games (over season)	H	56
				OH	
				HG	
				NC (various)	
Caccese et al., 2019 [12], USA	23 FY	OH, custom headband	Games (over season)	AI	Not specified
Chrisman et al., 2019 [13], USA	25 FEY	OH, behind ear	Games (over season)	AI	108
	21 MEY				81
Harriss et al., 2019 [14], Canada	36 FEY	OH, custom headband	Games (over season)	VH (*n* = 6)	≤720
				UH	
Miller et al., 2019 [15], USA	7 MEY	CM	Games and training (over season)	VH (*n* = 3)	103
				GH	
				OH	
Myer et al., 2019 [16], USA	11 FY	OH, behind ear	Play over season (regular game and training)	AI	14 games and 27 practices.
Rich et al., 2019 [17], USA	4 FEY	CM	Training (over season)	AI	9 practices
			Games (over season)		5 games
Sandmo et al., 2019 [18], Norway	6 MY	OH, inside ear	Training (over season)	H	12
				NH	
Mihalik et al., 2020 [19], USA	34 FY	OH, behind ear	Games (over season)	AI	2 seasons
			Training (over season)		
	41 MY		Games (over season)		
			Training (over season)		
Patton et al., 2020 [20], USA	23 FY	CM	Games (over season)	AI	18 games
	49 MY				23 games
Saunders et al., 2020 [21], USA	16 FY	OH, behind ear	Games (over season)	OH	417 measurements
				BH	
				HG	
				C	
	12MY			HH	229 measurements
				OH	
				BH	
				GH	
	16 FY		Training (over season)	HH	764 measurements
				OH	
				BH	
				GH	
				C	
	12 MY			OH	456 measurements
				BH	
				GH	
				C	
Filben et al., 2021 [22], USA	15 FY	CM	Play over season (regular game and training)	CH	72 practices and 24 games
				PH	
				AH	
Filben et al., 2021 [22], USA	6 FY	CM	Play over season (regular game and training)	H	34 practices and 18 games
	13 FY				54 practices and 20 games
Nelson et al., 2021 [23], USA	2 MY	OH, behind ear	Play over season (regular game and training)	AI	117 measurements
	3 MY				283 measurements
	5 MY				104 measurements
	2 MY				181 measurements
	1 FY				79 measurements
	3 FY				656 measurements
	9 FY				220 measurements
	3 FY				226 measurements
Nevins et al., 2019 [24], USA	8 MY	OH, behind ear	Play over season (regular game)	H	64 measurements
				BH	
				HG	
				HH	
				OH	
				NH (various)	
				NC	
	15 FY			H	135 measurements
				BH	
				HG	
				OH	
				NH (various)	
				NC	
Patton et al., 2021 [25], USA	18 MY	OH, custom headband	Play over season (regular game)	UH	60 measurements
				OH	
				F	
	27 MEY			UH	81 measurements
				OH	
				F	
Tomblin et al., 2021 [26], USA	14 FEY	CM	Play over season (regular game and training)	VH (*n* = 4)	32 practices and 34 games
				BH	
				NH (*n* = 2)	
				F (*n* = 2)	

Notes: Population: FEY = female early youth (i.e., ≤14 yrs old); FY = female youth (i.e., 15–21 yrs old); FM = female mixture (i.e., youth and adults); MEY = male early youth (i.e., ≤14 yrs old); MY = Male Youth (i.e., 15–21 yrs old); MM = male mixture. Sensor Location: OH = Outer head; CM = custom mouthpiece. Activity: H = Header; NH = Any non-header impact; F = Player fall; BH = Ball to head; GP = collision with goalpost; VH = Various header types; AI = Any head impact; NC = Type of head contact not clear; BH = Body to head contact; UH = Ball unintentionally hit head; HG = Head hit ground; PH = Passing header; CH = Clearing header; AH = Attacking header; HH = Head to head collision; OH = Other player collided with head; C = Combination of events. * Number of measurements refers to the product of the number of events and individuals monitored during the study. Since not all players participated on every event monitored this number can sometimes be calculated as an approximation (i.e., minimum or maximum value on the basis of the information provided within the study.

**Table 2:** Experimental studies reporting peak linear and rotational accelerations due to heading and other head impacts.

Reference	Population	Measurement Method	Scenario	Number of Measurements
Naunheim et al., 2000 [27], USA	UY	FH	Heading of a regulation size and weight soccer ball kicked from a distance of approximately 30 yards.	25
Lewis et al., 2001 [28], USA	3 MM	CM	Heading of a regulation size and weight soccer ball kicked from a distance of approximately 30 yards with and without a helmet	Not specified
Bayly et al., 2002 [29], USA	4 MA	OH, location not specified	Heading of a standard ball projected from a distance of 3 m using a mechanical soccer ball driver at speeds of 9 m/s and 12 m/s.	Not specified
Reed et al., 2002 [30], USA	6 MY & 1 FY	OH, headband	Heading the ball from standing position. Ball (size 4) lofted to the players with average speed 6.7 m/s from 3 m away by one of the camp′s coaches.	Not specified
Withnall et al., 2005 [31], USA	1 MA	BP	Heading of a ball projected from a soccer machine at a speed of 8 m/s from a distance of 5 m back to a target without a helmet and while wearing a helmet	5
Naunheim et al., 2003 [32], USA	4 MA	OH, headband	Heading a standard ball projected at 9 m/s from a distance of 6 m by a mechanical soccer ball driver (Soccer Tutor, Burbank, CA, USA). Driver was mounted 1.2 m from the ground.	12
			Heading a standard ball projected at 12 m/s from a distance of 6 m by a mechanical soccer ball driver (Soccer Tutor, Burbank, CA, USA). Driver was mounted 1.2 m from the ground.	12
Shewchenko et al., 2005 [33], Canada	7 MM	CM	Heading a ball projected to the player in speeds of either 6 or 8 m/s towards a target situated at 5.5 m away in a simulated passing scenario.	12
			Heading a ball projected to the player in speeds of either 6 or 8 m/s towards a target situated at 2.75 m away in a simulated ball control scenario.	3
			Heading a ball projected to the player in speeds of either 6 or 8 m/s as far away as possible from the player in a simulation of a clearing ball scenario	11
Shewchenko et al., 2005 [34], Canada	3MM	CM	Heading a ball projected to the player in speeds of either 6 or 8 m/s towards a target situated at 5.5 m away in a simulated passing scenario. Ball was a Fevernova Tri-lance of 444 g and 0.8 bar pressure (this is the baseline/common settings)	3
			Heading a ball projected to the player in speeds of either 6 or 8 m/s towards a target situated at 5.5 m away in a simulated passing scenario. Ball was a Fevernova Tri-lance of 444 g at a low pressure of 0.6 bar	3
			Heading a ball projected to the player in speeds of either 6 or 8 m/s towards a target situated at 5.5 m away in a simulated passing scenario. Ball was a Fevernova Tri-lance at a high pressure of 1.1 bar	3
			Heading a ball projected to the player in speeds of either 6 or 8 m/s towards a target situated at 5.5 m away in a simulated passing scenario. Ball was a Fevernova Junior 290 of low mass (351 g)	3
			Heading a ball projected to the player in speeds of either 6 or 8 m/s towards a target situated at 5.5 m away in a simulated passing scenario. Ball was a Fevernova Junior 350 of low mass (299 g)	3
Withnall et al., 2005 [35], Canada	5 MM & D	VM	Elbow to head impact during ball contention (the subject hits the manikin)	50
			Hand/wrist/forearm to head impact during ball contention (the subject hits the manikin)	50
Self et al., 2006 [36], USA	10 MY	OH, ear plugs	Heading a ball thrown from 50 m away by a soccer machine back to the direction it came from in a scenario simulating a header after goal kick	60
			Heading a ball thrown from 50 m away by a soccer machine in a scenario simulating a header after goal kick–i.e., with a redirection of the ball by 90 degrees from inflight path	60
Tierney et al., 2008 [37], USA	29 FY	CM	Heading of a ball projected from a JUGS soccer machine from a distance of 11 m under normal conditions (i.e., no headguard/helmet)	116
	15 MY		Heading of a ball projected from a JUGS soccer machine from a distance of 11 m under normal conditions (i.e., no headguard/helmet	60
	29 FY		Heading of a ball projected from a JUGS soccer machine from a distance of 11 m wearing a Full90 select performance headguard	116
	15 MY		Heading of a ball projected from a JUGS soccer machine from a distance of 11 m wearing a Full90 select performance headguard	60
	29 FY		Heading of a ball projected from a JUGS soccer machine from a distance of 11 m wearing a head blast soccer band	116
	15 MY		Heading of a ball projected from a JUGS soccer machine from a distance of 11 m wearing a head blast soccer band	60
Higgins et al., 2009 [38], USA	17 BY	CM	Heading of a ball projected from a JUGS soccer machine with a speed of 25 mph at an angle of 40° from a distance of 11 m (35 ft) to the participant.	170
Paris et al., 2010 [39], USA	1 MY	BP	Heading of a Baden 150 soccer ball, inflated to 55 kPa, thrown to the player by a JUGS Soccer Machine at four different launch speeds (no further data on speeds provided beside than one was 9.6 m/s and another 11.2 m/s). Four different distances form the machine were also applied (no data provided).	4–16
Dezman et al., 2013 [40], USA	8 MY	C	Heading of a ball served to the subjects by an investigator from 3 m away mimicking a soccer practice scenario of low ball velocity	40
	8 FY			40
Gutierrez et al., 2014 [41], USA	17 FY	OH, headband	Heading towards the front a ball thrown in to the player by a trained soccer player from 30 feet away. This was assumed as a simulated mimicking regular header drills they performed in practice.	51
			Heading towards the right a ball thrown in to the player by a trained soccer player from 30 feet away. This was assumed as a simulated mimicking regular header drills they performed in practice.	51
			Heading towards the left a ball thrown in to the player by a trained soccer player from 30 feet away. This was assumed as a simulated mimicking regular header drills they performed in practice.	51
Dorminy et al., 2015 [42], USA	10 MY 6 FY	CM	Heading of a ball projected from a JUGS soccer machine with a speed of 30 mph from a distance of 60 ft to the participant	25
			Heading of a ball projected from a JUGS soccer machine with a speed of 40 mph from a distance of 90 ft to the participant	25
			Heading of a ball projected from a JUGS soccer machine with a speed of 50 mph from a distance of 120 ft to the participant	25
Narimatsu et al., 2015 [43], Japan	11 MY	OH, headband	Heading of a ball projected using a JUGS soccer machine (JUGS Sports) from a distance of 9 m	55
Kawata et al., 2016 [44], USA	8 MY 2 FY	OH, base of skull	Heading of a ball (size 5, 8 psi inflation) projected using a JUGS soccer machine (JPS Sports, Tualatin, OR, USA) from a distance of 12 m at a speed of 11.2 m/s (which is similar to when soccer players make a long throw-in from the sideline to mid-field).	100
Wu et al., 2016 [45], USA	1 MA	CM	Heading a ball projected from a ball launcher (Sports Tutor, Burbank, CA, USA) with a speed of 7 m/s	10
		OH, behind ear		10
		OH, elastic skull cap		10
Caccese et al., 2017 [46], USA	42 MM	OH, elastic skull cap	Heading of a ball projected linearly using a JUGS soccer machine (JUGS, Tualatin, OR, USA) from a distance of approximately 12 m.	504
	58 FM			696
Caccese et al., 2018 [47], USA	42 MM 58 M	OH, elastic skull cap	Heading of a ball projected linearly using a JUGS soccer machine (JUGS, Tualatin, OR, USA) from a distance of approximately 12 m.	833
Hwang et al., 2017 [48], USA	8 MA 2 FA	OH, back of skull	Heading of a ball projected from a JUGs soccer machine from a distance of 12 m at a speed of 11.2 m/s directly back to the machine	100
Kuo et al., 2017 [49], USA	1 MA	CM	Heading a ball projected from a ball launcher (Sports Tutor, Burbank, CA, USA) with a speed of 7 m/s	14
Kuo et al., 2018 [50], USA	4 MA	CM	Heading a ball delivered using a ball launcher (Sports Tutor, Burbank, CA, USA) at speeds of up to 7 m/s which were expected to deliver an impact below 10 g	35
Sandmo et al., 2019 [18], Norway	6 MY	OH, ear canal	Heading exercises including finishing headers, redirectional headers, long direct headers, short direct headers, and headers from in-air duels.	431
			Nonheading exercises including shoulder-to-shoulder collisions, forceful shooting, redirectional running with maximal intensity, short straight sprinting with maximal intensity, falling abruptly forward on the ground and landing on out-stretched arms, and in-air duels without ball contact (losing the duel).	730
Nowak et al., 2020 [51], USA	16 MY 20 FY	OH, base of skull	Heading a ball projected from a distance of 40 ft by a mechanical JUGS with the ball traveling at 25 mph. The scenario simulates a long throw-in from the sideline to the midfield.	10
Smirl et al., 2020 [52], Canada	7 MA	OH, behind ear	Heading a ball projected from a distance of 25 m by a mechanical JUGS at a speed of 77.5 ± 3.7 km/h. Scenario was mimicking a heading following a corner kick.	40
Peek et al., 2021 [53], Australia	61 BY	OH, behind ear	Heading a ball thrown from a distance of 5 m by a trainer. Scenario was mimicking a heading following a thrown in. Ball was an Adidas starlancer size 5 of 432 g and inflated in 5 psi.	183
	61 BY		Heading a ball thrown from a distance of 5 m by a trainer. Scenario was mimicking a heading following a thrown in. Ball was a Heading-Pro size 4 of 255 g and inflated in 5 psi.	153
	51 BY		Heading a ball thrown from a distance of 5 m by a trainer. Scenario was mimicking a heading following a thrown in. Ball was a Deploy size 5 of 430 g and inflated in 5 psi.	75
	25 BY		Heading a ball thrown from a distance of 5 m by a trainer. Scenario was mimicking a heading following a thrown in. Ball was a Kickerball size 5 of 192 g and inflated in 5 psi.	183
Wahlquist and Kaminski., 2021 [54], USA	12 FEY	OH, headband	Heading a ball projected from a distance of 12.2 m by a mechanical JUGS at a speed 11.2 m/s (25 mph) and a 45-degree angle.	144
			Heading a ball projected from a distance of 12.2 m by a mechanical JUGS at a speed 11.2 m/s (25 mph) and a 45-degree angle. Participants received neck and core strengthening exercises.	144
Muller and Zentgraf., 2021 [55], Germany	15 MY	OH, headband	Heading a ball projected from a ball launcher (Freddie MAX, JofoSport, Czech Republic) with a speed of 9.6 m/s	90
			Heading a ball projected from a ball launcher (Freddie MAX, JofoSport, Vigantice, Czech Republic) with a speed of 10.8 m/s	90
	7 FY		Heading a ball projected from a ball launcher (Freddie MAX, JofoSport, Czech Republic) with a speed of 9.6 m/s	84
Austin et al., 2021 [56], UK	12 MA	C	Heading a ball projected from a distance of 4.7 m and 4 m above by a researcher back in 10 consecutive repeats	120
			Heading a ball projected from a distance of 4.7 m and 4 m above by a researcher back in 20 consecutive repeats	240
			Heading a ball projected from a distance of 4.7 m and 4 m above by a researcher back in 40 consecutive repeats	480
Victor Liberi., 1995 [57], USA	16 MY	OH, headband	Heading a dry ball projected by a mechanical leg from a distance of 18.5 m, with a speed of 15.5 m/s and an angle of 32 degrees	48
			Heading a wet ball projected by a mechanical leg from a distance of 18.5 m, with a speed of 15.5 m/s and an angle of 32 degrees	48
Peek et al., 2021 [58], USA	31 BEY	OH, behind ear	Heading a ball projected from a distance of ~5 m to the player by a trainer back to the direction of the throw. Participants received FIFA 11+ training	155
	31 BEY		Heading a ball projected from a distance of ~5 m to the player by a trainer back to the direction of the throw. No training received	155
	21 BEY		Heading a ball projected from a distance of ~5 m to the player by a trainer back to the direction of the throw. Participants received FIFA 11+ training and neck and core strengthening exercises	105
	21 BEY		Heading a ball projected from a distance of ~5 m to the player by a trainer back to the direction of the throw. No training received	105

Notes: Population: BEY = Both genders Early Youth (i.e., ≤14 yrs old); BY = Both genders Youth (i.e., 15–21 yrs); FEY = female early youth (i.e., ≤14 yrs old); FY = Female Youth (i.e., 15–21 yrs); FA = Female Adults; FM = Female Mixture of ages; MY = Male Youth (i.e., 15–21 yrs); MA = Male Adult; MM = Male Mixture of ages; D = Dummy; UY = Unspecified gender Youth (i.e., 15–21 yrs. Sensor Location: OH = Outer head; CM = custom mouthpiece, BP = bite plate; C = camera (motion capture system); VM = various places on manikin; FH = football helmet.

**Table 3:** Summary of studies reporting the effects of potential determinants of the acceleration of heading.

Reference	Year	Design	Determinant		Outcome	Evaluation Method	Results
			Name	Definition			
Sandmo et al. [18]	2019	EXP	Type of header	Finishing, redirectional, direct header long, non-heading duel, direct header short, non-heading events	PLA/ PRA	Descriptive graphical summary	PLA and PRA values higher in the order: finishing > redirectional > direct header, long > Non heading due l > Direct header short > Non heading events
Caccese et al. [47]	2018	EXP	Head mass (Kg), sternocleidomastoid (S.) strength (Kg), Heading technique	Head mass was estimated by multiplying body mass by the validated sex-specific head to total body mass percentage -S. strength was measured with a handheld dynamometer -Motion movement analysis related to extension and flexion during heading	PLA/ PRA	Linear regression	Increased head mass associated with decreased PRA. Higher S. strength associated with decreased PLA and PRA levels. No statistically significant difference observed for technique
Caccese et al. [46]	2017	EXP	Sex, player age	-Sex: male vs. female -Age: youth (12–14 yrs), high school (15–17 yrs), collegiate (18–24 yrs)	PLA/ PRA	Linear regression (MANOVA)	PLA and PRA levels on females were significantly higher than males. No statistically significant difference observed by age group
Dorminy et al. [42]	2015	EXP	ball speed	30 mph, 40 mph, 50 mph	PLA	Linear regression (MANOVA)	No systematic/significant differences observed
Dezman et al. [40]	2013	EXP	Sex, neck strength	-Sex: male vs. female -Neck strength: imbalance defined as the mean flexion strength minus mean extension strength measured with a spring-type clinical dynamometer	PLA/ PRA (Angular)	Spearman correlation	Mean neck strength imbalance was positively correlated (r = 0.5) with PLA and PRA, significant though only for the latter. No statistically significant differences observed between sexes.
Tierney et al. [37]	2008	EXP	Sex, head mass (Kg), head-neck segment length (cm), isometric strength (Kg)	Male vs. female	PLA	ANOVA, correlation analysis	Women exhibited greater PLA values than men. Head-neck mass and PLA were inversely correlated in scenarios with no helmet.
Self et al. [36]	2006	EXP	Type of header	Goal kick vs. corner kick	PLA	Non specified statistical hypothesis test	No statistically significant difference observed
Withnall et al. [35]	2005	EXP	Type of impact	Elbow-to-head vs. hand/wrist/forearm-to-head impact	PLA/ PRA	Descriptive comparisons	Differences between types of impact were small
Shewchenko et al. [60]	2005	EXP	Type of header	Passing vs. ball control vs. ball clearance	PLA/ PRA	Descriptive comparisons	Greater PLA and PRA for the controlling scenario vs. passing and clearing
Shewchenko et al. [33]	2005	EXP	Ball characteristics	ball mass, pressure, and construction characteristics	PLA/ PRA	Descriptive comparisons	A reduced ball mass and pressure appeared to relate to decreased PLA. An increase in ball pressure seemed to result in higher PLA and PRA.
Patton et al. [20]	2020	OBS	Sex	Male vs. female	PLA/ PRA	Descriptive comparisons, *t*-tests	No significant differences (*p* > 0.05) were found between females and males.
Miller et al. [15]	2019	OBS	Type of header	Kick, another header, throw, ground impact, headers received from a throw, headers from another header	PLA/ PRA	Linear mixed effect regression (random effect: athlete id)	Mean PLA and PRA values for kick higher than another header, or throw, and for PLA only for ground impact; headers received from a throw higher PLA to those from another header.
Harriss et al. [14]	2019	OBS	Type of header, Head impact location	-Type: pass in air, thrown in, deflection, punt, shot, goal kick, corner -Location: front, top, side,	HIF (for type of header only), PRA	Linear mixed effect regression (random effect: athlete and game id)	Type of header significant predictor of PRA. PRA: passes had higher values than deflections and thrown ins. Majority of impacts resulting from pass (41%) and throw ins (30%). Impact location significant predictor of PRA with level for top of head higher than frontal and side.
Rich et al. [17]	2019	OBS	Event type	Practice vs. Game	HIF, PLA/ PRA	Descriptive comparisons	HIF somewhat higher during practices compared to games. Small to no difference in median values of PLA.
Chrisman et al. [13]	2019	OBS	Age, Sex	-Age: 12 vs. 14 yrs - Sex: male vs. female	HIF (Age) PLA/ PRA (Sex)	Poisson regression (Age only) Descriptive comparisons (Wilcoxon rank sum tests)	Age effects were present but significant only for females (*p* = 0.02). Females sustained statistically significantly higher magnitude head impacts than males (*p* = 0.04).
Lamond et al. [10]	2018	OBS	Position, type of header, event type	-Position: DF vs. MD vs. FW vs. GK -Type of header: Clear vs. shot vs. pass vs. head-to-head vs. unintentional deflection -Event: practice vs. game	HIF, PLA	Linear mixed effect regression (random effect: number of impacts)	PLA: no difference across positions and events. DFs and MFs experience more impacts per game than FWs and GKs with DFs having the most. Average HIF per player per 10 events was larger in games than practices. Head-to-head impacts and unintentional deflections resulted in higher LA than purposeful headers. LA values for shots and clears were higher than passes.
Nevins et al. [11]	2018	OBS	Type of impact	Header vs. other type of impact (i.e., collision with player, collision with ground, player motion)	PLA/ PRA	ANOVA	Significantly greater PLA and PRA values for headers compared to player motion and false positive effects. PRA values higher for player motion.
Reynolds et al. [7]	2017	OBS	Sex, event type	-Sex: male vs. female -Event: practice vs. game	HIF, PLA/PLA	Negative binomial generalized estimate equation (GEE) models	Significantly more HI in games than practices. No other systematic/significant differences observed.
Reynolds et al. [8]	2017	OBS	Position, event type	-Position: DF vs. MD vs. FW vs. GK -Event: practice vs. game	HIF, PLA/ PRA	Descriptive comparisons	Average HIF per player somewhat higher in games than practices but with no considerable differences in PLA and PRA. HIF during game somewhat higher among DFs compared to GKs and/or FWs. Differences also observed in training: DFs had lower HIF numbers than players in other positions. PLA and PRA were lowest for GKs during games, and highest for GKs, DFs and MDs during practice.
Press et al. [6]	2017	OBS	Position, event type	-Position: DF vs. MD vs. FW vs. GK -Event: practice vs. game	HIF, PLA/ PRA (event type only)	Descriptive comparisons	Average HIF greatest for MDs, followed by DFs, FWs, and GKs. PLA and PRA higher in games than practices.
Lynall et al. [5]	2016	OBS	Position of play, event type, game half	-Position: DF vs. MF vs. FW and wide vs. central -Event: practice vs. game Game half: 1st vs. 2nd	HIF (position only), PLA/ PRA	chi-square test	Wide and MFs experienced more impacts than middle players and FWs and DFs, respectively. Practices had more impacts with high PLA/PRA than games and the same applied also for the 2nd half vs. the 1st.
Caccese et al. [3]	2016	OBS	Type of header	-Kick, goal kick, punt, corner kick, throw in, secondary header, bounce	PLA/ PRA	Linear mixed effect regression (random effect: number of impacts)	Goal kick and punt impacts resulted in higher PLA and PRA than kick impacts–bounce and secondary headers in lower.
Chrisman et al. [4]	2016	OBS	Sex	Male vs. female	HIF	Descriptive comparisons	Only 3 out of 7 female players performed headers whereas all 7 male players did so
McCuen et al. [2]	2015	OBS	Age	High school (14–18 yr) vs. collegiate (17–22 yr)	HIF, PLA/ PRA	Descriptive comparisons, *t*-tests	HIF somewhat higher among collegiate players compared with high school players. PLA values for impacts during games significantly lower for high school than collegiate players. No difference observed for PRA.
Hanlon and Bir. [1]	2012	OBS	Type of header, type of impact	-Side headers vs. front and back headers -Header vs. non-header (i.e., fall, unintentional ball-to-head, collision with player, collision with goalpost)	PLA/ PRA	ANOVA	PLA values for side headers higher than back headers. PRA values for side and front headers higher than back headers. |For type of impact, player collisions had the highest PLA values and falls the lowest.
Reed et al. [30]	2002	OBS	Event type	Practice vs. game	HIF	Descriptive comparisons	Average self-reported HIF higher for practices vs. games (adolescence game)
Filben et al. [22]	2021	OBS	play state, type of header, and outcome	-Play state: corner kick, goal kick, free kick, throw-in, drill, live ball -Type: pass, shot, or clearance -Outcome: successful vs. unsuccessful header	PLA/ PRA	Linear mixed effect regression (random effect: participant)	Headers during corner kicks, goal kicks, free kicks, and live balls had significantly greater PLA, PRA values than headers during drills. Successful headers had higher PLA values than unsuccessful ones.
Filben et al. [61]	2021	OBS	Age/level	-Collegiate vs. youth	PLA/ PRA	Linear mixed effect regression (random effect: participant)	Headers performed by collegiate players had significantly greater mean PLA, PRA values than youth players
Tomblin et al. [26]	2021	OBS	session type, position of play	-Game vs. practice -Position of play	PLA	Linear mixed effect regression (random effect: participant)	Practices were associated with higher PLA than games. Position had no effect
Nelson et al. [23]	2021	OBS	Sex, position of play, type of play	-Male vs. female -Position: DF vs. MF vs. FW vs. GP -Type of play: offensive vs. defensive vs. transition	PLA, PRA, HIF	ANOVA	Defenders had highest PLA vs. other positions. Females had higher PLA and PRA values than men. HIF was higher in males and in defenders
Liberi [57]	1995	EXP		Wet ball vs. dry ball	PLA	ANOVA	PLA values significantly higher when heading a dry ball
Nevins et al., [24]	2019	OBS	Sex, type of impact	-Male vs. female -Type: header vs. other type of impact (i.e., collision with player, collision with ground, collision with head, player motion)	HIF, PLA, PRA	Descriptive comparisons, chi-square and non-parametric median tests	Headers had the highest PLA and PRA values compared to other types of impacts. Males had significantly higher PLA and PRA median values for all impacts combined than females. Males also experienced higher values of HIF than females.
Patton et al. [25]	2021	OBS	Age, type of impact	-Age: 12–14 yrs vs. 14–16 yrs -Type: Ball to head vs. other type of impacts (i.e., collision with player, collision with ground (fall))	HIF, PLA	Descriptive comparisons, linear regression	Ball to head impacts significantly higher PLA values compared to other impact types. For 12–14 yr olds HIF highest for collision with other players. For 14–16 yr olds, HIF highest for ball to head impacts. Overall HIF per game highest for 14–16 yr olds.

EXP = experimental, OBS = Observational. PLA = Peak linear Acceleration, PRA = Peak rotational acceleration, HIF = Head Impact Frequency, DF = Defense, FW = Forward, MD = Midfield, GK = Goalkeeper. ANOVA = Analysis of the Variance.

## Data Availability

All data used in this review are contained in the Supplementary Tables.

## Supplementary Materials

The following supporting information can be downloaded at: https://www.mdpi.com/article/10.3390/ijerph19095488/s1, Table S1: Criteria for evaluating study quality and risk of bias in the reviewed studies, Table S2: Detailed information for observational studies reporting peak linear and rotational accelerations due to heading and other head impacts, Table S3: Detailed information for experimental studies reporting peak linear and rotational accelerations due to heading and other head impacts, Table S4: Results of the quality evaluation of the included studies.
