# Supplementary material for: A Systematic Review of Head Impacts and Acceleration Associated with Soccer
Source: Int J Environ Res Public Health. 2022 May 1;19(9):5488. doi: 10.3390/ijerph19095488 (PMC9100160; doi:10.3390/ijerph19095488)
Supplement: Supplementary file 1 [file ijerph-19-05488-s001.zip › ijerph-1659178-supplementary.pdf]

International Journal of
Environmental Research and Public Health

MDPI

Supplementary Table S1. Criteria for evaluating study quality and risk of bias in the reviewed studies.

Criteria	Score −1	Score 0	Score +1
Study design sufficiently described and free from potential bias	Study design poorly described or has potential biases (e.g. population selection)	Study design free from major biases but not described in detailed	Study design free from potential biases and described in detailed
Population characteristics (e.g. gender, number, age, experience) sufficiently described	Many details including gender and age missing	Some details of age, gender, number or experience missing	All relevant information are provided in detail
Population representativeness	-	Mannequins also included	Only humans included
Measurement methods adequately described	Measurement methods poorly described (i.e. important info like sampling device, location etc.) missing	Some but not all of the measurement methods described in detail	All measurement methods described in detail
Sample size adequately described	Neither the number of measurements nor the number of head impacts reported	Only the number of measurements or the number of impacts reported	Both number of measurements and impacts reported
Reporting of measurement results and their distribution	Only one of central values, ranges, min and/or max values reported	Central values reported with a single measure of spread	Central and spread values reported in detail (i.e. including ranges, standard deviations etc)
Head impacts confirmation	No confirmation	Confirmation by algorithm	Head impacts confirmed by observation (including in experiments)

Supplementary Table S2. Detailed information for observational studies reporting peak linear and rotational accelerations due to heading and other head impacts.

Reference, Country	Population	Measurement Method	Scenario	Activity	Number of Measurements	GM PLA (m/s²)	GM PRA (m/s²)	# of head Impacts per Player per Activity	# of head Impacts per Player/ Hour
Hanlon et al., 2012, USA	24 girls' youth soccer players ≤14 yrs age	HITS headgear snug fit to the back of the head With 6 (T 250 g) single-axis linear accelerometers (Analog Devices, Inc., Norwood) attached	Exhibition game (training)	Header (any type)	6 scrimmages of 35–60 min duration for 24 players	161.0	1468.4	2.0	1.3
				Any non-heading impact - e.g. player collisions with other players, player falls, collisions with goalposts, and unintentional collisions with the ball		181.5	1598.1	0.8	0.6
				Player fall (non heading)		130.0	953.2	0.3	0.2
				Unintentional ball-to-head		31304.6	1174.0	0.1	0.1
				collision with the goalpost		265.8	5179.5	0.0	0.0
McCuen et al., 2015, USA	29 female high school athletes (ages:14–18; mean: 15.7)	xPatch sensors (X2 Biosystems; Seattle, WA) affixed with adhesive patch behind the players right ear	Play over season (regular game)	Any type of head impact		302.5	6792.0	2.9	1.9
			Play over season (training)			295.7	6371.0	1.7	1.1

	14 female National Collegiate Athletic Association (NCAA) athletes (ages: 17–22; mean: 18.7)		Play over season (training)			301.1	6301.0	3.5	2.3
			Play over season (regular game)			312.8	6715.0	7.0	4.7
Caccese et al., 2016, USA	16 women's National Collegiate Athletic Association Division I collegiate soccer players playing in the regular season and excluding Goal keepers (19.6 ± 1.0 years, 60.3 ± 2.3 kg, 167.8 ± 3.8 cm)	Triax Smart Impact Monitor (SIM-G; Triax Technologies Inc., Norwalk, CT, USA) attached in a custom headband + video rec for verification	Play over season (regular game)	Goal kick (header off of a goal kick)	14 games (224 measurements max)	340.3	8576.4	0.1	0.1
				Punt (header in which the goal keeper punted the ball)		325.6	9122.2	0.1	0.1
				Kick (header in which another athlete on the field kicked the ball during game play, except for punts, goal kicks, and corner kicks, prior to the athlete heading the ball)		246.7	6580.9	0.7	0.5
				Corner kick (header off of a corner kick)		248.1	4829.9	0.1	0.0
				Throw-in (header off of a throw-in from the sidelines)		209.4	5318.5	0.3	0.2
				Secondary header (header in which another player headed the ball prior to the athlete heading the ball)		159.5	4352.2	0.1	0.1
				Bounce (header in which the ball made contact with the ground before the athlete headed the ball)		145.8	3411.2	0.2	0.1
Chrisman et al., 2016, USA	17 youth (7 female, 10 male) from 5 male and female soccer teams in the Seattle area participating in tournaments. Mean age was 12.6 yrs (SD = 1.0).	xPatch (X2 biosystems. com), mounted with single-use adhesive behind the ear + observation for impact verification	Play over a weekend tournament (3-6 games)	Overall (any type of head impact)	72 measurements	157.6		1.0	0.7
Lynall et al., 2016, USA	22 National Collegiate Athletic Association	xPatch system (X2 Biosystems, Seattle, WA, USA) affixed behind the ear with	Play over season (regular game)	Overall (any type of head impact)	18 games (252 measurements max)	109.8	1302.4	7.2	4.8

	(NCAA) Division I female soccer players of Mean age 19.1 yrs (SD = 0.1)	an adhesive patch	Play over season (training)		39 training events (858 measurements max)	102.6	1380.7	0.9	0.6
Press et al., 2016, USA	26 collegiate level female soccer players of mean (SD) age 19 yrs (1). Population included 3 goalkeepers, 9 midfielders, 5 forwards, and 9 defensive players	xPatch sensors (X2 Biosystems, Seattle, WA) affixed behind the ear with an adhesive patch + video rec	Play over season (regular game and training)	Header (Player intentionally headed the ball)	26 practices and 20 games (n = 916)	202.7	4567.5	1.7	1.1
				Up for header (Attempt to head the ball but exact contact unclear)		224.1	4776.4	0.1	0.1
				Fell down (Player fell down and head struck the ground)		150.6	3022.4	0.1	0.0
			Play over season (regular game and training)	Player to player contact (Player's head was struck by another player's body)		178.9	3361.1	0.0	0.0
				Unintentional ball to player contact (Player's head was unintentionally struck by the ball)		290.6	7105.7	0.0	0.0
Reynolds et al., 2017, USA	4 male soccer players part of a group of 14 players from 2 Division I soccer teams with mean age, of 20.4 yrs (SD = 1.3)	The xPatch (X2 Biosystems) on the skin covering their mastoid process	Play over season (regular game)						
	1 male soccer player part of a group of 14 players from 2 Division I soccer teams with mean age, of 20.4 yrs (SD = 1.3)		Play over season (regular game)	Goalkeeper	7	179.5	2589.8	20.9	16.7
	2 male soccer players part of a group of 14 players from 2 Division I soccer teams with mean age, of 20.4 yrs (SD = 1.3)		Play over season (regular game)	Defence	15	183.2	2700.8	46.5	37.2

	1 male soccer players part of a group of 14 players from 2 Division I soccer teams with mean age, of 20.4 yrs (SD = 1.3)	Play over season (regular game)	Midfield	2	137.3	2494.1	7.5	6.0
	4 male soccer players part of a group of 14 players from 2 Division I soccer teams with mean age, of 20.4 yrs (SD = 1.3)	Play over season (training)	Goalkeeper	50	170.6	2290.3	5.5	4.4
	2 male soccer players part of a group of 14 players from 2 Division I soccer teams with mean age, of 20.4 yrs (SD = 1.3)	Play over season (training)	Defence	93	171.4	2708.9	13.0	10.4
	4 male soccer players part of a group of 14 players from 2 Division I soccer teams with mean age, of 20.4 yrs (SD = 1.3)	Play over season (training)	Midfield	32	186.3	3337.2	7.7	6.2
	2 female soccer players part of a group of 7 players from 2 Division I soccer teams with mean age of 20.3 yrs (SD = 1.2)	Play over season (training)	Goalkeeper	42	203.9	3480.7	6.8	5.4
	2 female soccer players part of a group of 7 players from 2 Division I soccer teams with mean age of 20.3 yrs (SD = 1.2)	Play over season (training)	Midfield	36	192.7	3439.6	3.8	3.0

	3 female soccer players part of a group of 7 players from 2 Division I soccer teams with mean age of 20.3 yrs (SD = 1.2)		Play over season (training)	Forward	59	173.1	2394.6	5.4	4.3
Reynolds et al., 2017, USA	4 male college soccer players part of a group of 15 players with mean age of 20.2 (SD = 1.3) yrs. All volunteers from Division I teams	The xPatch (X2 Biosystems) on the skin covering their mastoid process	Play over season (training)	Goalkeeper	115 practices	189.0	3412.2	8.5	6.8
	6 male college soccer players part of a group of 15 players with mean age of 20.2 (SD = 1.3) yrs. All volunteers from Division I teams		Play over season (training)	Defence	197 practices	192.2	3170.4	7.7	6.1
	4 male college soccer players part of a group of 15 players with mean age of 20.2 (SD = 1.3) yrs. All volunteers from Division I teams		Play over season (training)	Midfield	125 practices	187.1	3121.0	4.5	3.6
	1 male college soccer players part of a group of 15 players with mean age of 20.2 (SD = 1.3) yrs. All volunteers from Division I teams		Play over season (training)	Forward	43 practices	167.7	2494.1	6.2	5.0

	1 male college soccer players part of a group of 15 players with mean age of 20.2 (SD = 1.3) yrs. All volunteers from Division I teams		Play over season (regular game)	Goalkeeper	2 games	134.4	2494	8.5	6.8
	1 male college soccer players part of a group of 15 players with mean age of 20.2 (SD = 1.3) yrs. All volunteers from Division I teams		Play over season (regular game)	Forward	7 games	195.2	2726.6	18.0	14.4
	3 male college soccer players part of a group of 15 players with mean age of 20.2 (SD = 1.3) yrs. All volunteers from Division I teams		Play over season (regular game)	Defence	19 games	134.4	2494.1	36.3	29.0
Bari et al., 2018. USA	23 female soccer athletes aged 14–17 years (mean = 15.9). All members of high school junior varsity or varsity teams and recruited from two local high schools over two seasons of play	xPatch (X2 Biosystems; Seattle, WA, USA) attached behind the right ear with adhesive patch	Play over season (regular game and training)	Overall (any type of head impact)	29 measurements over 1-2 different season.	294.2			
Lamond et al., 2018, USA	23 NCAA Division I female soccer student-athletes with mean age of 19.7 (SD = 1.2) years. Participants	Triax Smart Impact Monitor (SIM/-G; Triax Technologies, Norwalk, CT, USA) fitted in a headband, positioned around the nuchal line of the player + observations for	Play over season (regular game and training)	Pass (Trying to gain control of the ball through heading by either trapping or passing to a teammate. Used mostly by midfielders and forwards)	961	204.0		0.5	0.3

	included 3 goalkeepers, 3 defenders, 6 forwards = , and 11 midfielders	impact verification		Clear (deflection of the ball with no goal of gaining control simply to get the ball out of the area. Used mostly by defensive and midfield players)		291.4		0.1	0.1
				Shot (Heading the ball with the intention of scoring a goal. Used mostly by forwards)		293.9		0.0	0.0
				Head to Head (A collision with another's head (opponent, official, or teammate))		356.4		0.0	0.0
				Unintentional deflection (A head impact with the ball that was unexpected))		356.4		0.0	0.0
Nevins et a, 2018, USA	8 male high school soccer players with mean age of 16.9 (SD = 1.3) yrs	Xpatch sensors (X2 biosystems, Seattle, WA, USA) attached to the skin over the mastoid process + video analysis	Play over season (regular game)	Header (any type)	7 games (56 measurements)	321.7	6923.0	7.3	4.9
				Other player (Impact of contact with another player)		151.0	2644.0	2.6	1.7
				Ground (direct contact between head and ground)		146.1	2584	1.0	0.7
				Player motion (i.e. striking the ball and change of direction)		146.1	3913	0.3	0.2
				False impact (i.e. impact not verified by video)		139.3	2560.0	2.9	1.9
Caccese et a, 2019, USA	23 female soccer players with mean age 19.5 +-(SD = 5) yrs. Players included 6 forwards, 11 midfielders, 3 defenders, 3 goalkeepers	Triax Smart Impact Monitor (SIM-G; Triax Technologies Inc., Norwalk, CT, USA) embedded in elastic headbands positioned about the nuchal line.	Play over season (regular game)	Overall (any type of head impact)		154.1			
Chrisman et al., 2019, USA	25 female athletes from U12 and U14 select and premier teams of a Seattle youth soccer club. Players were aged 11 to 14 yrs old) and each played soccer for >6 years (85%)	The xPatch sensors (X2 Biosystems, Seattle, WA) attached to the skin over the mastoid process + observations for impact verification	Play over season (regular game)	Overall	108 games (108 measurements)	464.8		0.8	0.5

	21 male from U12 and U14 select and premier teams of a Seattle area youth soccer club. Players were aged 11 to 14 yrs and played soccer for >6 years (85%)				81 games (81 measurements)	329.5		0.5	0.3
Harriss et al., 2019, Canada	36 female soccer players with mean age of 13.4 (SD = 0.9) yrs, all members of 3 elite youth teams of the Ontario player development league	GFT2 fixed at headband + video recording for impact confirmation	Play over season (regular game)	Pass in air	60 regular season games (20 games per team) - i.e. <720 measurements	169.6		3.0	2.0
				Throw in		158.6		2.2	1.4
				Deflection		117.2		0.7	0.5
				Punt		156.9		0.6	0.4
				Shot		241.9		0.3	0.2
				Goal kick		186.6		0.3	0.2
				Corner		214.4		0.2	0.1
Miller et al., 2019, USA	7 athletes (two forwards, two midfielders, one defender, and two goalkeepers) enrolled in a local U-14 competitive soccer team. The players had a mean age of 13.4 (SD = 0.6) yrs old	Seven custom-instrumented mouthpieces featuring a tri-axial accelerometer and gyroscope embedded within a rigid retainer was used. Five of the mouthpieces used the sensor and battery components of an xPatch sensor (X2 Biosystems, Seattle, WA, USA) and the remaining two used sensor components developed by Stanford. Headers were confirm from video analysis by two independent researchers.	Play over season (regular game and training)	Header (Player intentionally headed the ball to either control it or after a header from a team player)	103 measurements (67 in practice and 36 in games)	77.5	658.8	2.4	1.6
				Kick (header in which another athlete on the field kicked the ball d prior to heading)		106.9	806.2	1.5	1.0
				Throw (Thrown from another athlete prior to heading)		97.1	677.3	3.4	2.2
				Ground (direct contact between head and ground)		81.4	709.9	0.1	0.1
				Other (contact between head and another player)		88.3	752.7	0.2	0.1
Myer et al., 2019, USA	11 female high school players aged 14–18 yrs that did not wear a collar	The xPatch sensors (X2 Biosystems, Seattle, WA) attached to the skin over the mastoid process	Play over season (regular game and training)	Overall	14 games and 27 practices.	193.4		15.9	10.6
Rich et al., 2019, USA	4 female athletes participating	A mouthpiece with an embedded accelerometer	Play over season (training)	Overall (any type of head impact)	9 practices	99.7	695.7	20.9	13.9

	in a local U14 soccer team	(Analog Devices, Norwood, MA, USA) and angular rate sensor (STMicroelectronics, Geneva, Switzerland)	Play over season (regular game)		5 games	99.0	637.0	3.3	2.2
Sandmo et al., 2019, Norway	6 male youth soccer players with mean age of 15.3 (SD = 0.3) yers; playing at the regional elite youth level in Norway during the 2017 season	MV1 sensor (MVTrak) worn in the left ear canal of subjects	Play over season (training)	Heading the ball	12 measurements across 2 sessions	180.7	12742.4	1.3	0.8
				Any non-heading impact-e.g. jumping, tackling, running with change of direction, and touching or losing the sensor.		31.0	260.7	168.7	112.4
Mihalik et al., 2020, USA	34 female aged 19.8 yrs (SD = 1.2). The study was performed at two Division 1 NCAA sites (Princeton University and the University of North Carolina at Chapel Hill)	The xPatch sensors (X2 Biosystems, Seattle, WA) attached to the skin over the mastoid process	Play over season (regular game)	Overall	2 seasons (i.e. yrs)	157.8	2618.8	10.0	6.7
			Play over season (training)			147.2	2225.5	10.0	6.7
	41 male aged 19.7 yrs (SD = 1.3). The study was performed at two Division 1 NCAA sites (Princeton University and the University of North Carolina at Chapel Hill).		Play over season (regular game)			179.2	3073.7	10.0	6.7
			Play over season (training)			152.0	2183.6	10.0	6.7
Patton et al., 2020, USA	23 adolescent female varsity players all in grades 9-12 high school	A triaxial accelerometer/gyroscope (SIM-G, Triax Tech, Norwalk, CT) mounted on a headband + video analysis	Play over season (regular game)	Overall (any type of head impact)	18 games	386.9		15.3	10.2
	49 adolescent male players (31 varsity and 18 junior varsity all in grades 9-12 high school).				23 games	412.0		45.3	30.2
Filben et al., 2021a, USA	15 female NCAA	A mouthpiece with an embedded	Play over season	Clearing	72 practices and 24 games	169.4	1529.6	0.9	0.1
				Passing		124.3	1003.6	0.1	0.1

	Division I players aged 19.8 yrs (SD = 1.24 years)	accelerometer (Analog Devices, Norwood, MA, USA) and angular rate sensor (STMicroelectronics, Geneva, Switzerland) custom-fit to each participant using 3D dental scans	(regular game and training)	Shot		136.9	1145.3	0.1	0.0
Filben et al., 2021b, USA	6 female players from one U15 team aged 15 yrs (SD = 0.11)	A mouthpiece with an embedded accelerometer (Analog Devices, Norwood, MA, USA) and angular rate sensor (STMicroelectronics, Geneva, Switzerland) custom-fit to each participant using 3D dental scans	Play over season (regular game and training)	Header (any type)	34 practices and 18 games	92.8	655	0.7	0.7
	13 female collegiate players from one NCAA Division 1 team aged 20.2 yrs (SD = 1.34)				54 practices and 20 games	220.6	2230	0.3	0.4
Nelson et al., 2021, USA	2 male NCAA Division III players aged 20.3 yrs (SD = 1.14)	xPatch sensor (X2 Biosystems Inc. Seattle, WA, USA) attached to the outer side of the head just below the ear	Play over season (regular game and training)	Goalkeeper	117 measurements	125.4	1222.6	0.3	0.2
	3 male NCAA Division III players aged 20.3 yrs (SD = 1.14)			Defense	283 measurements	203.9	4097.5	0.4	0.3
	5 male NCAA Division III players aged 20.3 yrs (SD = 1.14)			Midfield	104 measurements	183.1	3461.3	0.3	0.2
	2 male NCAA Division III players aged 20.3 yrs (SD = 1.14)			Forward	181 measurements	182.2	3343.9	0.9	0.6
	1 female NCAA Division III players aged 19.9 yrs (SD = 1.06)			Goalkeeper	79 measurements	144.5	2501.7	0.3	0.2
	3 female NCAA Division III players aged 19.9 yrs (SD = 1.06)			Defense	656 measurements	138.6	1818.7	0.2	0.2
	9 female NCAA Division III players aged 19.9 yrs (SD = 1.06)			Midfield	220 measurements	134.3	1717.8	0.1	0.1

	3 female NCAA Division III players aged 19.9 yrs (SD = 1.06)			Forward	226 measurements	135.1	1778.2	0.3	0.2
Tomblin et al., 2021, USA	14 female players from 2 local youth teams one of players aged 12–13 yrs and included 2 another of players aged 14–15 yrs	A mouthpiece with an embedded accelerometer (Analog Devices, Norwood, MA, USA) and angular rate sensor (STMicroelectronics, Geneva, Switzerland) custom-fit to each participant using 3D dental scans	Play over season (regular game and training)	Header while standing	32 practices and 34 games (924 measurments)	81.4	496.2	0.3	0.0
				Header while jumping		117.7	1142	0.1	0.0
				Body to body		42.2	238.7	0.2	0.0
				Body to head		138.3	2004.5	0.0	0.00
				Dives		42.2	418.9	0.3	0.1
				Falls		50.0	419.4	0.1	0.0
				Ball to body (unintentional)		41.2	241.6	0.0	0.0
				Ball to face (unintentional)		92.2	912.1	0.0	0.0
				Ball to head (unintentional)		118.7	2561.5		
Nevins et al., 2019, USA	8 high school males with mean age of 16.75 yrs (SD ± 1.09)	xPatch head impact sensors (X2 Biosystems, Seattle, WA, USA), mounted behind the right ear, just above the mastoid process + video analysis	Play over season (regular game)	Header (any type)	8 games (64 measurements)	325.6	6923	0.8	0.5
				Head to body		146.1	2251	0.0	0.0
				Head to ground		123.6	1732	0.0	0.0
				Head to head		216.7	2379	0.0	0.0
				Body to head		217.7	4404	0.0	0.0
				Body to body		1471	3286	0.3	0.2
				Body to ground		171.6	2805	0.1	0.1
				Player motion		104.9	2586	0.0	0.0
	15 high school females with mean age of 15.33 yrs (SD ± 1.01)			Header (any type)	9 games (135 measurements)	227.5	5515	0.6	0.4
				Head to body		176.5	5044	0.0	0.0
				Head to ground		134.4	3792	0.0	0.0
				Body to head		128.5	1875	0.2	0.1
				Body to body		134.4	2089	0.4	0.3
				Body to ground		133.4	1554	0.3	0.2
Patton et al., 2021,USA	18 male players aged between 14 and 16 years.	SIM-G head impact sensors (Triax technologies, Inc, Norwalk, CT, USA) were secured in a neoprene headband and positioned just above the greater occupital protuberance + Video analysis	Play over season (regular game)	Ball to head (unintentional)	4 games (60 measurements)	739.8		0.0	0.0
				Other player (Impact of contact with other player)		317.5		0.1	0.2
				Falls		298.5		0.1	0.2
	27 male players aged between 12 and 14 years			Ball to head (unintentional)	6 games (81 measurements)	555.7		0.1	0.1
				Other player (Impact of contact with other player)		291.5		0.0	0.1
				Falls		302.5		0.1	0.1
Saunders et al., 2020, USA	16 female NCAA Division III players with mean age 19.9 (SD = 1.1) yrs	Xpatch sensors (X2 biosystems, Seattle, WA) attached to the skin over the mastoid process + video analysis	Play over season (regular game)	Head to body	28 games (417 measurements)	201.3	3908.7	0.0	0.0
				Ball to head		232.0	4878.7	0.7	0.5
				Head to ground		169.7	2873.4	0.0	0.0
				Combination		186.3	3687.7	0.0	0.0
	12 male NCAA Division III players with mean age 20.3 (SD = 1.1) yrs			Head to head	21 games (229 measurements)	96.2	1412.6	0.0	0.0
				Head to body		132.6	1452.1	0.8	0.5
				Ball to head		172.6	2708.7	0.7	0.5
				Head to ground		139.0	1899.7	0.1	0.1
	16 female NCAA Division III players with		Play over season (training)	Head to head	52 practices (764 measurements)	134.3	1947.4	0.0	0.0
				Head to body		120.5	1345.0	0.0	0.0
				Ball to head		190.6	3133.3	0.6	0.4
				Head to ground		130.7	1244.7	0.0	0.0

mean age 19.9 (SD = 1.1) yrs	Combination		123.2	1381.9	0.0	0.0
12 male NCAA Division III players with mean age 20.3 (SD = 1.1) yrs	Head to body	44 practices (456 measurements)	118.2	1437.9	0.5	0.3
	Ball to head		137.3	1882.5	0.4	0.3
	Head to ground		142.6	2401.0	0.2	0.1
	Combination		147.1	3259.7	0.0	0.0

Supplementary Table S3. Detailed information for experimental studies reporting peak linear and rotational accelerations due to heading and other head impacts.

Reference	Population	Measurement Method	Scenario	Activity	Number of Measurements	GM PLA (m/s²)	GM PAA (m/s²)	Head Impacts per Player per Activity
Naunheim et al., 2000, USA	Elite level high school soccer players (no further data given)	Large Pro-Air II pneumatic and foam football helmet (Athletic Helmet, Inc., Knoxville, TN, USA) fitted with a triaxial accelerometer	A regulation size and weight soccer ball was kicked from a distance of approximately 30 yards.	Heading a regulation soccer ball kicked from 30 m away with no specific manner	25	502.3		
Lewis et al., 2001, USA	3 male volunteers aged between 16 and 30 yrs with high-school level soccer experience, familiar with the correct technique of ‘‘heading’’ a ball	Mouthpiece with PCB triaxial accelerometer (PCB Piezotronics, Inc., Depew, NY, USA)	Heading a ball kicked from a distance of approximately 30 yards with and without a helmet	Heading without a helmet a regulation soccer ball kicked from 30 m away with no specific manner		187.1		
				Heading with a helmet a regulation soccer ball kicked from 30 m away with no specific manner		74.9		
Bayly et al., 2002, USA	4 adult males between 25-36 years old who had played soccer in high school	Three tri-axial accelerometers (PCB Model 356B11) mounted to a headpiece fitted to the subjects head	Heading of a standard ball projected from a distance of 3 m using a mechanical soccer ball driver	Heading the ball back to the machine. Project speed ball by the machine of 9 m/sec		156.9	1263.5	
				Heading the ball back to the machine. Project speed ball by the machine of 12 m/sec		197.2	1427.6	
Reed et al., 2002, USA	7 adolescent players (6 males aged 13 and 1 female aged 16) of a youth soccer summer club.	Two PCB uniaxial accelerometers attached to a circumferential plastic band	Heading the ball from standing position. Ball (size 4) lofted to the players from 3 m away by one of the camp’s coaches.	Lofted ball to the head with an average speed of 6.7 m/sec		34.2		
Naunheim et al., 2003a, USA	A 30 year old male, having played competitive football in the past and continuing to play recreationally	2 orthogonal accelerometers (Endevco model 7264a–2000) mounted on a bite plate	Heading of a ball projected from a soccer machine from a distance of 5 m	Heading of a ball projected from a soccer machine from a distance of 5 m at a speed of 8.2 m/sec without a helmet.	5	72.9		

Naunheim et al., 2003b, USA	4 adult males between 25 and 36 yrs old who had played soccer in high school	Three triaxial accelerometers (PCB Model 356B11, PCB Piezotronics, Inc., Depew, NY) mounted on a polyethylene headpiece	Heading a ball projected from a distance of 6 m by a mechanical soccer ball driver (Soccer Tutor, Burbank, CA, USA) mounted 1.2 m from the ground.	Heading the ball back to the machine. Project speed ball by the machine of 9 m/sec	12	156.9	1263.5	3.0
				Heading the ball back to the machine. Project speed ball by the machine of 12 m/sec	12	197.2	1427.6	3.0
Shewchenko et al., 2005, Canada	7 players, aged 20–23 yrs old, all active participants in nonprofessional football and with 5–13 years' experience	A mouthpiece with attached accelerometers (Model 7264–2000 and 7302BM4; Endevco Corp., San Juan Capistrano, CA)	Heading a ball projected to the player in speeds of either 6 or 8 m/sec towards a target situated at 5.5 m away in a simulated passing scenario	Passing	12	143.9	1313.3	
			Heading a ball projected to the player in speeds of either 6 or 8 m/sec towards a target situated at 2.75 m away in a simulated ball control scenario	Controlling	3	188.9	1973.5	
			Heading a ball projected to the player in speeds of either 6 or 8 m/sec as far away as possible from the player in a simulation of a clearing ball scenario	Clearing	11	167.3	1504.1	
Shewchenko et al., 2005, Canada	3 players, aged 20–23 yrs old, all active participants in nonprofessional football and with 5–13 years' experience	A mouthpiece with attached accelerometers (Model 7264–2000 and 7302BM4; Endevco Corp., San Juan Capistrano, CA, USA)	Heading a ball projected to the player in speeds of either 6 or 8 m/sec towards a target situated at 5.5 m away in a simulated passing scenario with balls of different size and weight.	Passing using a Fevernova Tri-lance ball of 444 g and 0.8 bar pressure (this is the baseline/common settings)	3.00	156.0	1445.8	
				Passing using a Fevernova Tri-lance ball of 444 g and 0.6 bar pressure (low ball pressure)	3	138.5	1192.7	
				Passing using a Fevernova Tri-lance ball of 444 g and 1.1 bar pressure (high ball pressure)	3	141.0	1469.7	
				Passing using a Fevernova Junior 290 ball of 299 g (low mass ball)	3	139.5	1482.3	
				Passing using a Fevernova Junior 350 ball of 351 g (low mass ball)	3	171.0	1427.4	
Withnall et al. 2005, Canada	5 healthy and experienced football players, aged 18–30 years and a 50th	9 linear accelerometers in the so called 3-2-2-2 configuration attached to the dummy	Simulation of a head impact from an elbow (Elbow to head)	Elbow to head impact during ball contention where the player hits the dummy	50	188.6	1409.6	10.0

	percentile adult male Hybrid III dummy (Denton ATD Inc., Milan, OH, USA)		Simulation of as head impact from a hand, wrist or forearm (Hand/wrist/forearm to head)	Hand/wrist/forearm to head impact during ball contention where the player hits the dummy	50	187.1	1322.4	10.0
Self et al., 2006, USA	10 male volunteers from the U.S. Air Force Academy Varsity Soccer Team	Earplugs containing a ±500 g's Endevco 7269 tri-axial accelerometer	Heading a ball that is thrown from 50 m by a soccer machine to the player in a scenario simulating a header after a goal kick	Heading a goal kick back in the direction where the ball came from	60	28.5		
			Heading a ball that is thrown from 30 m by a soccer machine to the player in a scenario simulating a header after a corner kick or cross towards the goal	Heading a corner kick or cross towards the goal with a redirection of the ball by 90 degrees	60	31.8		
Tierney et al., 2008, USA	29 female volunteer soccer players with mean age of 19.5 (SD = 1.8) yrs with at least 5 years of heading experience	Custom-fit mouthpiece with triaxial accelerometer (model 35A; Endevco Corp, CA, USA) secured by dental wax.	Heading of a ball projected from a JUGS soccer machine from a distance of 11 m with and without a helmet, guard	Heading of a ball without a helmet	116	193.7		4.0
				Heading of a ball with a Full90 Select Performance Headguard (Full90 Sports Inc, San Diego, CA).	116	206.9		4.0
				Heading of a ball with a Head Blast Soccer Band (Head Blast Soccer Band Co, St Louis, MO).	116	204.5		4.0
	15 male volunteer soccer players with a mean age of 20.3 (SD = 2.9) yrs with at least 5 years of heading experience			Heading of a ball without a helmet	60	171.8		4.0
				Heading of a ball with a Full90 Select Performance Headguard (Full90 Sports Inc, San Diego, CA, USA).	60	143.0		4.0
				Heading of a ball with a Head Blast Soccer Band (Head Blast Soccer Band Co, St Louis, MO, USA).	60	149.2		4.0
Higgins et al., 2009, USA	17 college soccer players with a mean age of 20.9 yrs (SD = 1.2)	A custom-fit mouthpiece with a triaxial accelerometer (model 35A; Endevco Corp, San Juan Capistrano, CA, USA) secured on it	Heading of a ball projected from a JUGS soccer machine with a speed of 25 mph at an angle of 40° from a distance of 11 m (35 ft) to the participant.	Heading of a projected ball projected back to a target located between the participant and the JUGGs	170	236.3		10.0

Paris et al., 2010, USA	One 18-year-old male in good physical condition	Custom acrylic mouth guard with an AD ADXL250 Dual Axis Accelerometer	Heading of a Baden 150 soccer ball, inflated to 55 kPa, thrown to the player by a JUGS® Soccer Machine at different speeds and from different distances were also applied (no data provided) simulating a proper heading scenario	Proper heading while standing with player heading a projected Baden 150 soccer ball, inflated to 55 kPa,	4 -16 (depending on number of distances tested by speed)	<333		
Dezman et al., 2013, USA	8 male college-level soccer players from Division I and II programs	A 14-camera Vicon MX3 Motion Capture System (Vicon Motion Systems, Los Angeles, CA, USA).	Heading of a ball served to the subjects by an investigator from 3 m away mimicking a soccer practice scenario of low ball velocity	Heading of a low velocity ball	40	85.7	753.0	5.0
	8 female college-level soccer players from Division I and II programs				40	96.4	791.8	5.0
Gutierrez et al., 2014, USA	17 female varsity high school soccer players from the same school team with a mean age of 15.9 (SD = 0.9)yrs	A custom headband, fitted with a triaxial accelerometer (Type 8690C5, Kistler Instrument Corp., Amherst, NY, USA)	Heading a ball thrown to the player by a trained soccer player from 30 feet away. This was assumed as a simulated mimicking regular header drills they performed in practice	Forward header	51	53.4		
				Left header	51	64.9		
				Right header	51	58.2		
Dorminy et al., 2015, USA	10 male and 6 female collegiate players in Division I with a mean age of 20.4 (SD = 0.24) yrs), with ≥5 yrs of experience	A custom mouthpiece with triaxial accelerometer fitted (model 35A; Endevco Corp, San Juan Capistrano, CA, USA)	Heading of a ball projected from a JUGS soccer machine with different speeds and from different distances	Heading back a ball projected with a speed of 30 mph from a distance of 60 ft to the participant	25	335.1		5.0
				Heading back a ball projected with a speed of 40 mph from a distance of 90 ft to the participant	25	472.7		5.0
				Heading back a ball projected with a speed of 50 mph from a distance of 120 ft to the participant	25	492.6		5.0
Narimatsu et al., 2015, Japan	11 male high school soccer players with a mean age of 16.8 yrs	A triaxial accelerometer (Wireless Sensor Module 50G, Logical Product Corporation) put in a headband	Heading of a ball projected using a JUGS soccer machine (JUGS Sports) from a distance of 9 m	Heading of a ball projected from a JUGS soccer machine back in free style and no mouth guard (i.e. as the players would choose to do)	55	270.4		5.0

Kawata et al., 2016, USA	8 males and 2 females with a mean age of 20.7 (SD = 1.2)yrs, all current soccer team members with at least 5 years of heading experience	A triaxial accelerometer (Gforce Tracker Inc, Markham, ON, USA) taped below the external occipital protuberance	Heading of a ball projected using a JUGS soccer machine (JPS Sports, Tualatin, OR, USA) from a distance of 12 m at a speed of 11.2 m/s (which is similar to when soccer players make a long throw-in from the sideline to mid-field).	Heading of a ball projected from a JUGS soccer machine back to a target located at ~5 m from the player	100	133.2		10.0
Wu et al., 2016, USA	One 26 year-old male.	A mouth guard equipped with an accelerometer (ST H3LIS331), a gyroscope (Invensense ITG-3500A), and a sensing module	Heading a ball projected from a ball launcher (Sports Tutor, Burbank, CA, USA) with a speed of 7 m/sec	Heading back a ball projected from a launcher	10	89.2	696.4	
		An xPatch G2 sensor (X2Biosystems, Inc.) adhered to the mastoid process			10			
		An elastic skull cap (Reebok) equipped with an accelerometer (ST H3LIS331), a gyroscope (Invensense ITG-3500A), and a sensing module			10			
Caccese et al., 2017, USA	42 active soccer male players across youth (n = 8, 12–14 yrs old), high school (n = 14, 15–17 yrs old) and collegiate (n = 20, 18–24 yrs old) ages. No goalkeepers and all with at least 1 year of playing competitively	A triaxial accelerometer/gyroscope (SIM-G, Triax Tech, Norwalk, CT, USA) secured to the back of the head with an elastic cap.	Heading of a ball projected linearly using a JUGS soccer machine (JUGS, Tualatin, OR, USA) from a distance of approximately 12 m	Heading a projected back to a target located at ~2 m away from the player	504	258.7	2080.5	12.0
	58 active soccer female players across youth (n = 18, 12–14 yrs old), high school (n = 19, 15–17 yrs old) and collegiate (n = 21, 18–24 yrs old) age. No goalkeepers and all with at least 1 year of playing competitively				696	381.4	3118.6	12.0

	26 active soccer youth (12–14 years old) players (8 males, 18 females). No goalkeepers, all at least 1 yr of playing competitively				312	377.6	3026.0	12.0
	33 active soccer high school (15–17 years old) players (14 males, 19 females). No goalkeepers, all at least 1 yr of playing competitively				396	328.5	2745.0	12.0
	41 active soccer collegiate (18–24 years old) players (20 males, 21 females). No goalkeepers, all at least 1 yr of playing competitively.				492	341.3	2792.0	12.0
Caccese et al., 2017, USA	100 soccer players including 42 males and58 females with mean age of17.1 (SD = 3.5) and 13.3 (SD = 3.0) yrs respectively. No goalkeepers and at least 1 yr experience.	A triaxial accelerometer/gyroscope (SIM-G, Triax Tech, Norwalk, CT, USA) secured to the back of the head with an elastic cap.	Heading of a ball projected linearly using a JUGS soccer machine (JUGS, Tualatin, OR, USA) from a distance of approximately 12 m	Heading a projected back to a target located at ~2 m away from the player	833	322.1	2614.5	10.0
Hwang et al., 2017, USA	8 males and 2 females with a mean age of 21 (SD = 1.2) yrs. All active members of an organized soccer team (i.e., collegiate, intramural, club), and with at least 5 years of soccer playing experience	A triaxial accelerometer (Gforce Tracker Inc., Markham, ON, USA) secured on the back of the head with pre-wrap and tape	Heading a ball projected from a distance of 12 m by a JUGS soccer machine (JPS Sports, Tualatin, OR, USA) at speeds of 11.2 m/sec	Heading of projected ball directly back to the machine	100	135.5		10.0
Kuo et al., 2017, USA	1 healthy male aged 26 yrs old	A custom mouth guard fitted with a triaxial accelerometer (H3LIS331DL) and a triaxial gyroscope (ITG3500A)	Heading a ball projected from a ball launcher (Sports Tutor, Burbank, CA, USA) with a speed of 7 m/sec	Heading a ball projected from a ball launcher back towards launcher	14	81.4	847.0	14.0

Kuo et al., 2018, USA	4 male subjects with an average age of 25.5 (SD = 2.1) yrs	Custom made mouth guard containing an inertial measurement unit (i.e. triaxial linear accelerometer and gyroscope)	Heading a ball delivered using a ball launcher (Sports Tutor, Burbank, CA, USA) at speeds of up to 7 m/s which were expected to deliver an impact below 10 g	Perform a mild heading	35	57.5	597.8	
Sandmo et al., 2019, Norway	6 male youth players with mean age of 15.3 (SD = 0.3) yrs at Norway's 2017 regional elite youth level	MV1 sensor (MVTrak) worn in the left ear canal of subjects	Structured training including 5 heading and 6 non-heading exercise drills typical for soccer	Heading exercises including finishing headers, redirectional headers, long direct headers, short direct headers, and headers from in-air duels	431	122.0	7345.9	71.0
				Non-heading exercises including shoulder-to-shoulder collisions, forceful shooting, redirectional running with maximal intensity, short straight sprinting with maximal intensity, falling abruptly forward on the ground and landing on out-stretched arms, and in-air duels without ball contact (losing the duel)	730	43.6	875.3	121.0
Nowak et al., 2020, USA	36 subjects (16 males, 20 females) with least 5 years of experience and a mean age of 20.4 (SD = 1.7) yrs	A triaxial accelerometer (SIM-G; Triax Technologies Inc) secured in a headband and positioned below the external occipital protuberance (inion)	Heading a ball projected from a distance of 40 ft by a mechanical JUGS with the ball traveling at 25 mph. The scenario simulates a long throw-in from the sideline to the midfield.	Heading following long throw-in from the sideline to the midfield.	10	312.832135	3300	10.0
Smirl et al., 2020, Canada	7 male soccer players with mean age 24.1 (SD = 1.5) yrs. All with >5 yrs of experience in senior or university playing level	An xPatch (X2 Biosystems; Seattle, WA) accelerometer placed over the right mastoid process	Heading a ball projected from a distance of 25 m by a mechanical JUGS at a speed of 77.5 ± 3.7 km/h. Scenario was mimicking a heading following a corner kick.	Heading following a corner kick	280	397.6	8057.1	40.0
Wahlquist and Kaminski, 2021, USA	12 female youth soccer players in the Under 12	A Triax SIM-G (Triax Technologies, Norwalk, CT, USA) head impact	Heading a ball projected from a distance of 12.2 m at	Heading of projected ball directly back to the machine	144	216.6	2432.7	12

	division from a local youth soccer club	sensor with a triaxial accelerometer and triaxial gyroscope inserted into a headband secured to the back of the participant's head	11.2 m/s (25 mph) and at a 45-degree angle by a mechanical JUGS.	Heading of projected ball directly back to the machine, Participants received neck and core strengthening exercises.		219.9	2110.3	12
Peek et al., 2021a, Australia	61 male and female players registered with a with mean age (mean) 14.5 yrs	Players wore a close-fitting latex 'swim cap' that housed an IMU (AX6™, Axivity™, Newcastle, UK) with a tri-axial accelerometer and a tri-axial gyroscope	Heading a ball thrown from a distance of 5 m by a trainer. Ball was an Adidas starlancer size 5 of 432 g and inflated in 5 psi.	Passing after a throw in	183	60.4		3
	61 male and female players registered with a with mean age (mean) 14.5 yrs		Heading a ball thrown from a distance of 5 m by a trainer. Ball was a Heading-Pro size 4 of 255 g and inflated in 5 psi.		183	107.7		
	51 male and female players registered with a with mean age (mean) 14.5 yrs		Heading a ball thrown from a distance of 5 m by a trainer. Ball was a Deploy size 5 of 430 g and inflated in 5 psi.		153	77.0		
	25 male and female players registered with a with mean age (mean) 14.5 yrs		Heading a ball thrown from a distance of 5 m by a trainer. Ball was a Kickerball size 5 of 192 g and inflated in 5 psi.		75	148.3		
Muller and Zentgraf, 2021, Germany	15 male players from local soccer clubs with mean age of 16.5 yrs (SD = 1.1)	Player wore an accelerometer (Noraxon, Scottsdale, AZ, USA) attached above the Os occipitale using a headband.	Heading a ball projected from a ball launcher (Freddie MAX, JofoSport, Czech Republic) from a distance of 10–15 m a and with a speed of 9.6 m/sec	Heading projected ball back	90	81.3		12
			Heading a ball projected from a ball launcher (Freddie MAX, JofoSport, Vigantice, Czech Republic) from a distance of 10–15 m and with a speed of 10.8 m/sec		90	90.8		
	7 female players from local soccer clubs with mean age 16.5 yrs (SD = 0.3)		Heading a ball projected from a ball launcher (Freddie MAX, JofoSport, Czech Republic) from a distance of 10-15 m and with a speed of 9.6 m/sec		84	103.1		

Liberi Victor, 1995, USA	16 collegiate male varsity players with a mean age of 16 yrs, excluding goalkeepers. All with Division I college experience	A triaxial quartz beam accelerometer (3.4 g) mounted on an adjustable nylon headband that was fitted tightly over the inion.	Heading a dry ball projected from a mechanical leg from a distance of 18.5 m, with a speed of 15.5 m/sec and an angle of 32 degrees	Heading projected ball back	48	217.7		6
			Heading a wet ball projected from a mechanical leg from a distance of 18.5 m, with a speed of 15.5 m/sec and an angle of 32 degrees		48	201.9		6
Austin et al., 2021, UK	12 males with mean age of 23.7 yrs (SD± 4.8). All with a history of head injury within the last year and recreational experience of heading	Impact Kinematics were collected using a 10-camera three dimensional motion capture system (3D MoCap; Vicon T40S, Oxford, UK) sampling at 1000 Hz.	Heading a ball projected from a distance of 4.7 m and 4 m above by a researcher back in 10 consecutive repeats	Heading projected ball a low velocity ball	120	153.6	2229.2	10
			Heading a ball projected from a distance of 4.7 m and 4 m above by a researcher back in 20 consecutive repeats		240	129.7	1924.5	20
			Heading a ball projected from a distance of 4.7 m and 4 m above by a researcher back in 40 consecutive repeats		480	140.6	2038.2	40
Peek et al., 2021b, Australia	31 players (17 males, 14 females) aged 14.35 yrs (SD = 0.29) registered with one high-level youth football club	Players wore a close-fitting latex 'swim cap' that housed an IMU (AX6™, Axivity™, Newcastle, UK) with a tri-axial accelerometer and a tri-axial gyroscope	Heading a ball projected from a distance of ~5 m to the player by a trainer back to the direction of the throw. No training received	Passing after a throw in	155	126.016		
			Heading a ball projected from a distance of ~5 m to the player by a trainer back to the direction of the throw. Participants received FIFA 11+ training		155	111.2074		5
	21 players (14 males, 7 females) aged 14.95 (SD = 0.21) registered with one high-level youth football club		Heading a ball projected from a distance of ~5 m to the player by a trainer back to the direction of the throw. No training received		105	113.07		

		Heading a ball projected from a distance of ~5 m to the player by a trainer back to the direction of the throw. Participants received FIFA 11 + training and neck and core strengthening exercises	105	107.38	

Supplementary Table S4. Results of the quality evaluation of the included studies. Evaluation is performed on the basis of the criteria summarised in Supplementary Table 1.

Reference	Design	Study Design Sufficiently Described and Free from Potential bias	Population Characteristics (e.g. Gender, Number, Age, Experience) Sufficiently Described	Population Representativeness	Measurement Methods Adequately Described	Sample Size Adequately Described	Reporting of Measurement Results and their Distribution	Head Impacts Confirmation	Comments	Overall Score
Bari et al., 2018	OBS	1	0	1	1	1	1	0		5
Bayly et al., 2002	EXP	1	0	1	1	−1	0	1		3
Caccese et al., 2019	OBS	1	1	1	1	0	0	0		4
Caccese et al., 2017	EXP	1	1	1	1	1	0	1		6
Caccese et al., 2017	EXP	1	1	1	1	1	0	1		6
Caccese et al., 2016	OBS	1	1	1	1	0	0	1		5
Chrisman et al., 2016	OBS	1	1	1	1	1	0	1		6
Chrisman et al., 2019	OBS	1	1	1	1	1	0	1		6
Dezman et al., 2013	EXP	1	1	1	1	1	0	1	Headers are performed on a standing position which may not be 100% representative of real conditions	6
Dorminy et al., 2015	EXP	1	1	1	1	1	1	1		7
Filben et al., 2021	OBS	1	1	1	1	0	1	1		6
Filben et al., 2021	OBS	1	1	1	1	0	1	1		6
Gutierrez et al., 2014	EXP	1	1	1	1	1	0	1		6
Hanlon et al., 2012	OBS	1	0	1	1	0	1	1		5
Harriss et al., 2019	OBS	1	1	1	1	0	0	1		5

Higgins et al., 2009	EXP	1	1	1	1	1	0	1	Headers are performed on a standing position which may not be 100% representative of real conditions	6
Hwang et al., 2017	EXP	0	1	1	1	1	0	1	Header methodology not described in detail	5
Kawata et al., 2016	EXP	1	1	1	1	1	1	1	Headers are performed on a standing position which may not be 100% representative of real conditions	7
Kuo et al., 2018	EXP	0	0	1	1	1	1	1		5
Kuo et al., 2017	EXP	0	1	1	1	1	0	1	Header methodology not described in detail	5
Lamond et al., 2018	OBS	1	1	1	1	1	1	1		7
Lewis et al., 2001	EXP	1	1	1	1	-1	0	1		4
Lynall et al., 2016	OBS	1	1	1	1	1	0	0		5
McCuen et al., 2015	OBS	1	1	1	1	-1	0	-1		2
Mihalik et al., 2020	OBS	1	0	1	1	0	0	0		3
Miller et al., 2019	OBS	1	1	1	1	1	1	1		7
Myer et al., 2019	OBS	1	1	1	1	1	1	-1		5
Narimatsu et al., 2015	EXP	1	0	1	1	1	0	1		5
Naunheim et al., 2000	EXP	0	-1	1	1	1	0	1		3
Withnall et al., 2005	EXP	0	1	1	1	1	1	1	Only one subject included	6
Naunheim et al., 2003	EXP	1	0	1	1	1	0	1		5
Nelson et al., 2021	OBS	1	1	1	1	1	1	1		7
Nevins et al., 2018	OBS	1	1	1	1	1	1	1		7
Nowak et al., 2020	EXP	1	1	1	1	1	1	1		7
Paris et al., 2010	EXP	0	0	1	1	1	-1	1		3
Patton et al., 2020	OBS	1	0	1	1	1	0	1		5

Peek et al., 2021	EXP	1	1	1	1	1	0	1		6
Peek et al., 2021	EXP	1	1	1	1	1	-1	1		5
Press et al., 2016	OBS	1	1	1	1	1	1	1		7
Reed et al., 2002	EXP	0	1	1	1	-1	1	1		4
Reynolds et al., 2017	OBS	1	1	1	1	1	1	0		6
Reynolds et al., 2017	OBS	1	1	1	1	1	1	0		6
Rich et al., 2019	OBS	1	0	1	1	1	1	1		6
Sandmo et al., 2019	EXP / OBS	1	1	1	1	1	1	1		7
Saunders et al., 2020	OBS	1	1	1	1	1	1	1		7
Self et al., 2006	EXP	0	-1	1	1	1	1	1		4
Shewchenko et al., 2005	EXP	1	1	1	1	1	1	1		7
Shewchenko et al., 2005	EXP	1	1	1	1	1	1	1		7
Smirl et al., 2020	EXP	1	1	1	1	1	0	1		6
Tierney et al., 2008	EXP	1	1	1	1	1	0	1		6
Tomblin et al., 2021	OBS	1	0	1	1	1	1	1		6
Wahlquist and Kaminski, 2021	EXP	1	1	1	1	1	0	1		6
Withnall et al., 2005	EXP	0	1	0	1	1	0	1	Only one subject involved	4
Wu et al., 2016	EXP	0	0	1	1	1	-1	1	Only one subject involved	3
Nevins et al., 2019	OBS	1	1	1	1	1	0	1		6
Liberi Victor., 1995	EXP	1	0	1	1	1	0	1		5
Patton et al., 2021	OBS	1	0	1	1	1	1	1		6
Austin et al., 2021	EXP	1	1	1	1	1	0	1		6
Muller and Zentgraf., 2021	EXP	1	1	1	1	1	0	1		6

EXP = Experimental, OBS = Observational,
